# Taming the In-Basket—How Two Simple Tools Reduced Portal Message Volume in an Academic Internal Medicine Clinic

**DOI:** 10.1007/s11606-025-09478-7

**Published:** 2025-04-15

**Authors:** Nicole Hadeed, Jessica Ameling, James Henderson, Matthew Bucala, Yvette Salamey, Jennifer Meddings

**Affiliations:** 1 Division of General Medicine, Department of Internal Medicine, University of Michigan Medical School, Ann Arbor, MI USA; 2 Institute for Healthcare Policy and Innovation, University of Michigan, Ann Arbor, MI USA; 3 Department of Pediatrics and Communicable Diseases, University of Michigan Medical School, Ann Arbor, MI USA; 4 Center for Clinical Management Research, VA Ann Arbor Healthcare System, Ann Arbor, MI USA

**Keywords:** Patient portal messaging, In-basket burden, Primary care, Workflow efficiency, Quality improvement

## Abstract

**Background:**

Patient portal messaging has emerged as a critical tool in primary care, particularly during the COVID-19 pandemic, facilitating asynchronous communication between patients and providers. The surge in portal messages during the pandemic has exacerbated work overload and burnout among primary care providers.

**Objective:**

To enhance primary care clinic portal message workflow efficiency by identifying barriers through surveys and interviews, then implementing interventions to address challenges and streamline processes.

**Design:**

We used a pre- and post-intervention with concurrent control design to evaluate this quality improvement project. The project was conducted at an Internal Medicine ambulatory clinic site within a large academic medical center in the Midwest.

**Participants:**

The quality improvement project involved primary care physicians, registered nurses, licensed practical nurses, medical assistants, and patient services associates managing patient portal messages in primary care clinics.

**Intervention:**

Interviews and surveys assessed workflow practices, perceptions, and gaps. Interventions included developing “Best Practice Standards” and “Routing Guide” documents and restructuring staffing with dedicated time for message management.

**Main Measures:**

The co-primary outcomes were the volume of portal messages per physician clinical full-time equivalent (cFTE) and the proportion of message encounters with physician involvement. The secondary outcome was the proportion of messages sent to multiple recipients (“carbon copy” messages).

**Key Results:**

The intervention site showed a 16% reduction in messages per physician cFTE monthly (RRR, 0.84; 95% CI, 0.75–0.94) and a 65% decrease in “carbon copy” messages (RR, 0.35; 95% CI, 0.31–0.39) compared to controls. Physician involvement in messages remained unchanged at the intervention site but increased 8% at control sites. Pre-intervention interviews identified workload, process, training, and stress barriers. Post-intervention, staff noted improvements from role clarification and dedicated message time.

**Conclusion:**

Targeted interventions can reduce portal message burden and improve workflow efficiency in primary care by implementing standardized protocols and clarifying roles.

**Supplementary Information:**

The online version contains supplementary material available at 10.1007/s11606-025-09478-7.

## INTRODUCTION

Patient portal messaging, a web-based tool allowing patients to securely send messages to their healthcare providers, has become an important and highly utilized tool in primary care. The patient portal was designed to improve patient and physician communication, quality of care, and access to care by offering patients a platform to asynchronously discuss preventative, acute, and long-term healthcare needs with physicians and clinical staff.^1–8^ This tool was increasingly utilized during the SARS-CoV-2 (COVID-19) pandemic. Primary care physicians (PCPs) and ambulatory sites relied on remote forms of communication, including portal messaging, to address health concerns when it was not possible or unsafe to bring patients into the office.^9–12^ In primary care, this resulted in a 157% increase in patient messages during the peak of the pandemic compared to pre-pandemic levels.^1,2,13–15^

The volume of portal messages in primary care has remained significantly higher than pre-pandemic levels, adding to usual physician and staff responsibilities and exacerbating work overload and burnout, a well-identified issue even before the pandemic.^16–21^ Patient-initiated portal messages are one component of the physician’s “in-basket” in the electronic health record (EHR), but account for a large proportion of time spent by physicians on in-basket tasks and significantly correlate with burnout.^18,22^ With primary care facing the brunt of this meteoric rise in in-basket work, the sustainability of primary care is under threat without creative solutions to counteract the burden of this additional work.^23,24^

While the increase in portal message use and its impact on patient care, work overload, and burnout are well-described, the effectiveness of solutions to improve portal messaging processes is understudied. Many proposed solutions for the in-basket, such as those by the American Medical Association, include improving efficiencies within the EHR and creating a team-based infrastructure to tackle in-basket tasks.^25,26^ Just as critical are strategies to operationalize and maintain the team-based approach to in-basket tasks within a clinic unit. Many healthcare institutions support this using dedicated staff for in-basket management, distributing message load across disciplines, and creating communication protocols for staff; ^3,27,28^ however, questions remain on how best to implement this team-based approach for efficient task management and communication.

We report on a quality improvement initiative aimed at enhancing portal message workflow in a primary care clinic. The initial phase involved surveying and interviewing physicians and staff to understand existing workflows, perceptions, and gaps related to portal messages. We then developed and implemented documents clarifying team responsibilities and best practices to improve communication efficiency. Using a pre-post design with concurrent control clinics, we evaluated the hypothesis that this would reduce portal message volume and improve physician and staff satisfaction through difference-in-differences analysis.

## METHODS

This study was conducted at one Internal Medicine ambulatory clinic site within a large academic medical center, where faculty and resident physicians treat primary care patients. Two clinics served as control sites. The intervention clinic comprises faculty with diverse roles, including primarily clinical, as well as research, education, and administrative roles. Staff support both a faculty and a resident clinic at the intervention site. The control clinics comprised primarily clinical faculty. The resident clinics were not included in the pilot. During the pre-intervention phase, informal site visits (“Gemba Walks”) were conducted at the intervention and control sites, observing clinic workflows on portal message handling to gain insights on positives and pain points. The project team also reviewed results from a prior project examining clinic workflows and standardized practices at a different site. Pre-intervention interviews informed intervention design. Survey and EHR data comparisons (pre to post, intervention to control) measured intervention effectiveness, while post-intervention interviews provided context. The project timeline is shown in Appendix Section A.

### Interviews

We used pre-intervention interviews to understand the current workflow used to manage portal messages and solicit opinions on interventions. Separate physician and staff interview guides were developed (Appendix Section B). The main interview topics were (1) Process of handling portal messages in the clinic; (2) “Prework” completed before forwarding portal messages to the physician; (3) Training, education, and protocols around portal message management; (4) Ideas for improvement; and (5) Stress and burnout related to portal messages.

Post-intervention interviews measured the implementation of in-basket messaging interventions (Appendix Section B). Topics included participants’ impressions of portal messaging best practices, the usefulness of the intervention tools, observed changes in messaging practices, impacts of staffing shortages, and the effectiveness of dedicated time for medical assistants. Sustainability, stress, burnout, and future considerations were also addressed.

Physicians, registered nurses, licensed practical nurses, and medical assistants involved in managing portal messages were invited via email to participate in interviews. Pre-intervention individual interviews were conducted via video conferencing (August–October 2022), while post-intervention group interviews were held in-person (July–August 2023). A qualitatively trained project team member (JA) led the semi-structured interviews using role-specific guides. Interviews were audio recorded, transcribed, and de-identified.

### Intervention Tools and Logistics

**Figure 1:**
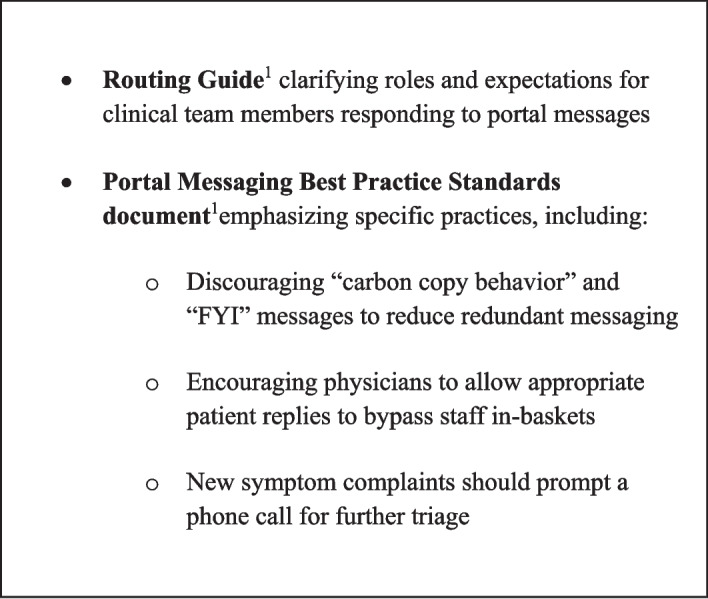
Key intervention components to optimizing in-basket routing (see Appendix Section C)

Based on pre-intervention findings, two documents were developed to address barriers such as lack of standardized protocols, confusion around roles, and inefficient routing practices: (1) “Best Practice Standards” for message handling/routing; and (2) a “Routing Guide” clarifying physician and staff roles for correctly routing patient messages (Appendix Section C). Both documents were disseminated through staff meetings, huddles, emails, and hard copies. By providing documents emphasizing role clarification and routing practices, we aimed to reduce message traffic between team members and decrease redundant routing and messages received by physicians. Additionally, staffing was restructured to pull one staff member to address portal messages on one morning per week to facilitate dedicated in-basket time on high-volume days. Key aspects of the intervention are highlighted in Fig. 1.

### Measures

#### Surveys

Pre-intervention interviews informed the development of custom surveys for physicians (20 questions) and staff (22 questions) to assess portal management pain points and satisfaction (Appendix Section D). The surveys covered portal messaging impact, burdensome message types, training, routing practices, prework expectations, satisfaction, stress, and burnout. Surveys were administered electronically at both intervention and control sites via Qualtrics in November 2022 (pre-intervention) and July 2023 (post-intervention).

#### Electronic Health Record Metrics

The co-primary outcomes were the volume of portal messages sent to all clinic inboxes relative to physician cFTE and the proportion of portal message encounters involving physician involvement (a message sent or received by a physician). The secondary outcome measure was the instances of “carbon copy behavior,” where a single message was sent to multiple recipients. Additional exploratory metrics are described in Appendix Table E.1.

#### Data Analysis

Interview transcripts were analyzed using qualitative content analysis with matrix analysis to identify themes.^29,30^ We used descriptive analysis to analyze survey results. We described continuous variables from the surveys using means and interquartile ranges and computed differences between the intervention and control sites using two-sample, homogenous *t*-tests. We described categorical variables from the surveys using percentages and computed differences in percents using large-sample *z*-tests without continuity correction.

We used difference-in-differences regression models to compare EHR metrics changes between the intervention and control clinics. Numerators and denominators for each metric were aggregated by period and intervention status. Rates were modeled using negative binomial models, and rate and relative rate ratios were computed by exponentiating coefficients. Difference in differences (DID) were calculated using average marginal effects.^31^ Estimates are reported with 95% CIs and considered significant when excluding the null. Analysis was performed in R v4.3.3 from September 2023 to April 2024.

### Ethical Review and Project Integrity

The Institutional Review Board deemed the protocol “not regulated” as it was a Quality Assurance/Quality Improvement project. We reported this project following the SQUIRE 2.0 quality improvement guidelines.^32^

## RESULTS

### Interviews

#### Pre-intervention Interviews

The pre-intervention phase involved 12 interviews with 14 participants (6 physicians, 4 RNs, 1 LPN, 3 MAs) from three primary care clinics (Table 1). Key themes included overwhelming portal workload, lack of standardized pre-work procedures, inadequate training/protocols leading to confusion, need for written protocols to streamline workflow, and pervasive staff stress/burnout due to high message volume.

**Table 1:** Pre-intervention Interview Themes and Representative Quotes

Theme	Representative quote
Inconsistent portal message routing practices	“I get letter requests for the simplest darn things that nobody has done…You know, the patient wrote a portal message saying I saw you; I was sick, I was off from X day and I’m going back this day. I need a letter. Again, you could have anybody do that, but it isn’t done generally. It’s done by the MD.” (PCP)
Lack of standardized pre-work for portal messaging	“But somebody has to review them [portal messages] and determine where they go and, in our clinic, that’s where it is right now, and it’s a heap of work. It hasn’t been working but it hasn’t been addressed or changed…” (RN)
Staff feels stress and burnout due to lack of training around portal messaging	“No (regarding if they had training). And if I can be really, really, really brutely honest, I like to say that my training is kind of like they took me on a ship in the middle of the ocean at 60 foot and dropped me in and then told me to find the island.” (LPN)
Providers feel stress and burnout due to insufficient “pre-work” of portal messages	“So, what’s most frustrating to me is getting messages with very little information, asking for what recommendations to do next when I feel as if it would be most helpful for any new clinical symptom to be triaged, and actually have a good understanding or the best one can of the surrounding situation.” (PCP)
Support for developing portal messaging standard or protocol	“I don’t know that we have anything written out, which would probably be helpful in prioritizing and who should be getting what and what you can do to expedite it to the physician and what leg work you can do.” (MA)

*MA*, medical assistant; *LPN*, licensed practical nurse; *RN*, registered nurse; *PCP*, primary care physician

#### Post-intervention Interviews

During the post-intervention phase, 6 interviews (3 joint) with 14 participants (6 physicians, 6 RNs, 1 clinic manager, 1 administrative manager, and 4 MAs) provided insights into implementation and lessons learned (Table 2). The routing document clarifying roles emerged as a highly valuable component, while staff considered guidance on avoiding “FYI” messages too vague. Dedicating extra staff for portal work temporarily helped when short-staffed but was unsustainable for right-sizing workloads. Notable staffing shortages during April–May perpetuated inefficiencies and heightened stress levels in portal message management.

**Table 2:** Post-intervention Interview Themes and Representative Quotes

Theme	Representative quote
Uncertainty Around Implementation	“I think people are inertial and if it’s worked and you don’t see it all the time, you just don’t think about it. And if you have a small practice too, a lot of the inefficiencies don’t bother you as much.” (PCP)
Routing Document Significance	“I like this document. I feel like this document helps when you are orienting a new staff member to know the roles. It also says to you who gets what, where does this go.” (MA)
Divergent Perspectives on Best Practices Document	“I thought it was excellent and even though—and I don’t know that we have entirely realized the benefit of it yet. I think it took time to really start to see the benefit once people get used to following the best practices.” (PCP)
Challenges in Dedicated MA Inbox Management Role	“Mondays being—it’s hard on Mondays. That’s a hard day to do just a half-day because you felt like you didn’t really—you personally felt like you didn’t get anything accomplished…” (MA)
Impact of Staffing Shortage	“Well, it’s pretty tough to route things to a team that is composed of one person who doesn’t have enough time to do their job.” (PCP)

*MA*, medical assistant; *LPN*, licensed practical nurse; *RN*, registered nurse; *PCP*, primary care physician

### Surveys

Eight staff members completed the pre-intervention survey (67% overall response rate) and 11 completed the post-intervention survey at the intervention site (92% overall response rate), with RNs representing 12% and 27% of respondents, LPNs 9% and 6%, MAs 50% and 55%, and Patient Service Associates (PSAs 38% and 9%, respectively). At control sites, there were 29 participants who completed pre-intervention surveys and 30 participants who completed post-intervention surveys, with RNs representing 26% and 23% of respondents, LPNs 17% and 6%, MAs 26% and 20%, and PSAs 40% and 40%, respectively (Appendix Table G. 1–12).

At the intervention site, we surveyed 9 physicians and 2 advance practice providers (APPs) before the intervention (73% response rate for physicians) and 5 physicians after (42% response rate). At control sites, we surveyed 15 physicians before the intervention (68% response rate) and 13 physicians after (59% response rate) (Appendix Tables G. 1–12).

Among the survey items, clarity of expectations for clinical team members in responding to portal messages showed the most notable change. Combining staff and physician responses, mean scores at the intervention site increased from 2.7 to 3.5 on a 6-point Likert scale (1 = Strongly Agree, 5 = Strongly Disagree, 6 = N/A or Don’t Know), indicating improved clarity. Control sites showed a smaller change, from 2.8 to 2.7.

### EHR Metrics

From September 2022 to August 2023, we analyzed a total of 342,990 in-basket messages, encompassing both intervention and control clinics. This includes messages sent by patients to the clinic, internal clinic communications, and messages sent from clinic staff to patients as part of an ongoing conversation. It excludes one-way outgoing messages from the clinic to patients (such as appointment reminders or general announcements). These messages were associated with 87,504 portal encounters representing 31,510 unique patients. Included patients had an average age of 55.9 years, with 59.7% female, 3.1% Hispanic, 91.8% non-Hispanic, 0.2% American Indian/Alaskan Native, 7.4% Asian, 8.2% Black, 78.4% White, and 4.0% Other race (Table 3).

**Table 3:** Patient Characteristics

Characteristic	Overall	Intervention site			Control sites			Intervention vs control
	*N* (%)	Pre, *N* (%)	Post, *N* (%)	Difference, % (95% CI)	Pre, *N* (%)	Post, *N* (%)	Difference, % (95% CI)	Difference, % (95% CI)
Total patients	31,510^1^ (100)	3967 (100)	3585 (100)	-	20,358 (100)	18,166 (100)	-	-
Age^2^, mean (IQR)	55.9 (43.5–69.1)	53.7 (37.0–68.8)	53.8 (37.7–68.6)	0.1 (− 0.7 to 0.9)	56.5 (44.7–69.2)	56.3 (44.6–69.1)	− 0.2 (− 0.5 to 0.2)	− 2.6 (− 3.1 to − 2.2)
Female	18,827 (59.7)	2075 (52.3)	1918 (53.5)	1.2 (− 1.1 to 3.4)	12,568 (61.7)	11,228 (61.8)	0.1 (− 0.9 to 1.0)	− 8.9 (− 10.1 to − 7.7)
Race								
American Indian or Alaska Native	67 (0.2)	13 (0.3)	15 (0.4)	0.1 (− 0.2 to 0.4)	40 (0.2)	33 (0.2)	− 0.0 (− 0.1 to 0.1)	0.2 (0.0 to 0.3)
Asian	2407 (7.6)	318 (8.0)	270 (7.5)	− 0.5 (− 1.7 to 0.7)	1500 (7.4)	1280 (7.0)	− 0.3 (− 0.8 to 0.2)	0.6 (− 0.1 to 1.2)
Black or African American	2164 (6.9)	395 (10.0)	378 (10.5)	0.6 (− 0.8 to 2.0)	1282 (6.3)	1166 (6.4)	0.1 (− 0.4 to 0.6)	3.9 (3.2–4.6)
White	25,153 (79.8)	2938 (74.1)	2666 (74.4)	0.3 (− 1.3 to 0.8)	16,536 (81.2)	14,788 (81.4)	0.2 (− 0.6 to 1.0)	− 7.1 (− 8.2 to − 6.0)
Other^2^	1288 (3.9)	227 (5.7)	195 (5.4)	− 0.3 (− 1.3 to 0.8)	694 (3.4)	649 (3.6)	0.2 (− 0.2 to 0.5)	2.1 (1.6–2.7)
Unknown^3^	491 (1.6)	76 (1.9)	61 (1.7)	− 0.2 (− 0.8 to 0.4)	306 (1.5)	306 (1.5)	− 0.1 (− 0.4 to 0.1)	0.4 (0.0–0.7)
Ethnicity								
Hispanic	932 (3.0)	165 (4.2)	147 (4.1)	− 0.1 (− 1.0 to 0.8)	543 (2.7)	469 (2.6)	− 0.1 (− 0.4 to 0.2)	1.5 (1.0–2.0)
Non-Hispanic	29,010 (92.1)	3678 (92.7)	3350 (93.4)	0.7 (− 0.4 to 1.9)	18,687 (91.8)	16,756 (92.2)	0.4 (− 0.1 to 1.0)	1.1 (0.4–1.7)
Unknown^4^	1568 (5.0)	124 (3.1)	88 (2.5)	− 0.7 (− 1.4 to 0.1)	1128 (5.5)	941 (5.2)	− 0.4 (− 0.8 to 0.1)	− 2.6 (− 3.0 to − 2.1)

^1^Unique patients. Patients may overlap sites and may appear in both pre and post periods. ^2^At project start, September 1, 2022, and excluding 21 patients with missing birth dates. ^3^Other includes Multi, Other, and 14 Native Hawaiian and Other Pacific Islander included here to prevent cell sizes < 11 to protect patient privacy. ^4^Unknown includes Choose Not to Disclose, Unknown, and 10 missing values. ^5^Unknown includes Choose Not to Disclose, Unknown, and 116 missing values

Of the 342,990 messages received, 72.4% were addressed to message pools (a role-based in-basket managed by multiple staff members in a single role), 24.4% to physicians, and 3.6% to advanced practice providers and others. As a single message can be sent to more than one recipient, these 342,990 received messages were generated by 296,362 sent messages. The sent messages originated from patients (50.6%), medical assistants (13.2%), nurses (17.3%), physicians (8.6%), and other clinical, administrative, or technical staff (10.3%). Denominators for reported rates are in Appendix Table G.2.

Our co-primary outcomes were the volume of portal messages sent to all clinic inboxes relative to physician cFTE per month and the proportion of portal message encounters with physician involvement. In the 3 months prior to the intervention (September–December 2022), there were 1342 (95% CI 1269–1420) messages per cFTE per month at the intervention site and 1067 (95% CI 1010–1127) messages per cFTE per month at control clinics. Following a 4-month implementation and washout period (January to April 2023), in the 4-month post period (May–August 2023), there were fewer messages per cFTE per month at both the intervention site (954 msg/cFTE/m; 95% CI, 902–1010) and control sites (935 msg/cFTE/m; 95% CI, 885–988). The reduction was significantly larger at the intervention clinic in both relative (RRR, 0.84; 95% CI, 0.75–0.94) and absolute (DID, 219.6; 95% CI, 109.7 to 329.6) terms (Table 4). Denominators for reported rates are in Appendix Table G.2. The proportion of portal message encounters involving physician involvement showed no change (RR, 0.99; 95% CI, 0.97–1.02) at the intervention site, with rates of 60.3% (95% CI, 59.3–61.3%) pre-intervention and 59.9% (95% CI, 58.9–61.0%) post-intervention. Control sites had a lower percent of encounters with physician involvement in both pre (46.9%; 95% CI, 46.4–47.4%) and post (50.6%; 95% CI, 50.1–51.1) intervention periods but also an 8% (RR 1.08; 95% CI, 1.06–1.10) relative increase.

**Table 4:** Relative Change for Portal Messaging Metrics Post-intervention Compared to Pre-intervention

	Intervention site			Control sites			
Measure (per month)	Pre, mean (95% CI)	Post, mean (95% CI)	RR (95% CI)	Pre, mean (95% CI)	Post, mean (95% CI)	RR (95% CI)	RRR (95% CI)
Messages sent per cFTE^1^	1342 (1269–1420)	954 (902–1010)	0.71 (0.66–0.77)	1103 (1044–1166)	935 (885–988)	0.85 (0.78–0.92)	0.84 (0.75–0.94)
% encounters with physician involvement^2^, %	60.3 (59.3–61.3)	59.9 (58.9–61.0)	0.99 (0.97–1.02)	46.9 (46.4–47.4)	50.6 (50.1–51.1)	1.08 (1.06–1.10)	0.92 (0.90–0.95)
% “Carbon Copy” messages (with more than 1 recipient), %	4.4 (4.1–4.6)	1.5 (1.4–1.7)	0.35 (0.31–0.39)	1.1 (1.0–1.1)	1.0 (0.9–1.0)	0.90 (0.83–0.97)	0.38 (0.33–0.44)

*cFTE*, clinical full time equivalent; *RR*, risk ratio; *RRR*, relative risk ratio

^1^Excludes messages sent to patients

^2^Excluding physician-triaged encounters

The secondary outcome, instances of “carbon copy behavior,” showed a significant 65% reduction (RR, 0.35; 95% CI, 0.31–0.39) at the intervention clinic, decreasing from 4.4% of messages before the intervention to 1.5% after. This substantial decrease indicates that the intervention was effective in reducing redundant messaging.

Exploratory metrics showed a larger reduction in messages per encounter at the intervention site (22%; RR, 0.78; 95% CI, 0.77–0.80) compared to control sites (13%; RR, 0.87; 95% CI, 0.86–0.88). Messages sent directly to physicians decreased significantly more at the intervention site (26%; RR, 0.74; 95% CI, 0.67–0.83) than at control sites (RRR, 0.86; 95% CI, 0.74–1.00). However, encounters with physician-sent messages increased by 39% (RR, 1.39; 95% CI, 1.29–1.50) at the intervention site, from 11.90% to 16.50%, but remained below control clinics (23.00% pre, 24.60% post). This increase was confounded by physician triage of initial patient messages due to a severe staff shortage at the intervention site during the pilot, with a significant increase in physician-initiated encounters (RR, 3.58; 95% CI, 3.00–4.27) compared to stable rates in controls (RR, 0.93; 95% CI, 0.81–1.08), a limitation of the study. Full exploratory metrics are in Appendix Table G.3.

Exploratory metrics showed a 22% reduction in the number of messages per encounter at the intervention site (RR, 0.78; 95% CI, 0.77–0.80) compared to a 13% decline at control sites (RR, 0.87; 95% CI, 0.86–0.88). Additionally, messages sent directly to physicians decreased by 26% (RR, 0.74; 95% CI, 0.67–0.83) at the intervention site, a significantly larger reduction compared to control sites (RRR, 0.86; 95% CI, 0.74–1.00). This reduction in messages sent directly to physicians is a positive outcome, as it suggests that the implemented interventions, such as the “Best Practice Standards” and “Routing Guide,” were effective in redirecting messages to the appropriate staff members. Full details on exploratory metrics can be found in Appendix Table G3.

## DISCUSSION

Our findings, in line with existing research, underscore the growing prevalence of portal messaging in primary care and its notable association with burnout among physicians and healthcare staff, thereby underscoring the imperative for targeted interventions to alleviate these issues.^33–35^ Our pre-intervention interviews and surveys revealed that the lack of standardized protocols and clear role delineation in portal management led to confusion, inefficiencies, and increased workload for team members. These challenges, which have been reported in prior studies across different healthcare settings, ^25,36–38^ highlight the need for interventions that establish clear guidelines and processes to improve the efficiency and effectiveness of portal management in primary care. Figure 1 summarizes the most helpful aspects of our intervention targeting this.

In this pre/post-intervention quality improvement project, we implemented simple, locally controlled, cost-neutral interventions that markedly reduced redundant routing practices and led to fewer portal messages circulating the clinic to be received by physicians. In the current landscape of healthcare, the increase in asynchronous care using the patient portal has expanded and entwined workflows across clinic team members. Our initiative emphasizes the importance of clarity in workflow and its efficacy in decreasing burden across a clinic. Formalizing role delineation for in-basket tasks was of particular importance in improving efficient teamwork. Outlining practices in our “Best Practice Standards” included avoidance of duplicitous routing and “FYI” messages and encouraged physicians to allow appropriate patient replies to bypass staff in-baskets, all of which encouraged sensitivity to the work of other team members and improved team cohesiveness.

It was evident during the intervention that while innovation can create substantial gains, such efforts are significantly hampered when staff shortages reach a critical threshold. In the case of the intervention, this shortage shifted more non-physician tasks to physicians and significantly increased the burden and stress on remaining staff. This impact could not be circumvented by pulling a staff member from a different role on high-volume days, as it left another area to manage with less. It was also evident that the shortage led to certain shortcuts in established workflows out of necessity, which created lingering bad habits even when staffing numbers were restored.

Our intervention approach has been substantiated by our outcome evaluation, demonstrating its effectiveness in diminishing portal message volume and enhancing operational efficiency.^39–41^ This approach, although inspired by strategies observed in other healthcare institutions, is distinct in its implementation, providing nuanced insights into the challenges specific to primary care clinics. A point of further exploration will involve strategies to maintain the workflow shifts and behavioral change in this intervention. Maintenance of change will likely require a different approach to the initial creation and implementation of changes we describe and is important to explore over a longer period. Our intervention period spanned 4 months, and this question was out of scope for our study.

We acknowledge several limitations. (1) The intervention overlapped with a period of a particularly significant shortage of staff who directly manage portal messages, leading to physicians more directly managing their messages. It is possible this involvement contributed to the reduction in total message traffic. Multiple avenues to attempt to correct for this were considered and ultimately deemed impossible to accomplish. However, we do not expect this to have contributed to less carbon copy behavior, which was one significant source of internal message traffic and volume. (2) The intervention site was also the clinic site of the principal investigator (PI) of the project. The PI’s presence, although not directly involved in data collection, may have influenced the intervention’s success through increased personal investment, clinic engagement, and real-time feedback, which might be less replicable at a site lacking a dedicated physician champion.

## CONCLUSION

Through this work, we identified a gap in routing standards for managing portal messages. By providing documents emphasizing role clarification and routing practices, we successfully reduced message traffic between clinic team members, leading to a decrease in redundant routing and messages received by physicians. We propose other systems to take a similar approach in identifying local gaps in training and role clarification to enhance in-basket management, decrease volume, and bolster well-being.

## Supplementary Information

Below is the link to the electronic supplementary material.

Supplementary file1 (DOCX 254 KB)
