# Supplementary material for: Taming the In-Basket—How Two Simple Tools Reduced Portal Message Volume in an Academic Internal Medicine Clinic
Source: J Gen Intern Med. 2025 Apr 15;40(16):4022–9. doi: 10.1007/s11606-025-09478-7 (PMC12686222; doi:10.1007/s11606-025-09478-7)
Supplement: Supplementary file 1 — Supplementary file1 (DOCX 254 KB) [file 11606_2025_9478_MOESM1_ESM.docx]

**Appendix**

Table of Contents

Section A. **Section A.** Timeline Highlighting Key Phases of the Project .…………….………….1

[Section B. Pre- and Post-Intervention Interview Guides.](file:///C:\Users\mbucala\AppData\Local\Temp\MicrosoftEdgeDownloads\9f63b71f-41a4-4825-8568-160a000d76ca\11606_2023_8415_MOESM1_ESM.docx#_Toc143273403) 2-8

[Section C. Best Practices and Routing Guides 9-13](file:///C:\Users\mbucala\AppData\Local\Temp\MicrosoftEdgeDownloads\9f63b71f-41a4-4825-8568-160a000d76ca\11606_2023_8415_MOESM1_ESM.docx#_Toc143273407)

[Section D. Pre- and Post-Intervention Staff and Provider Surveys 14-28](file:///C:\Users\mbucala\AppData\Local\Temp\MicrosoftEdgeDownloads\9f63b71f-41a4-4825-8568-160a000d76ca\11606_2023_8415_MOESM1_ESM.docx#_Toc143273411)

[Section E. Definitions for Portal Messaging Metrics 29](file:///C:\Users\mbucala\AppData\Local\Temp\MicrosoftEdgeDownloads\9f63b71f-41a4-4825-8568-160a000d76ca\11606_2023_8415_MOESM1_ESM.docx#_Toc143273412)

[Table E.1. Complete Definitions for Portal Messaging Metrics from the EHR 29](file:///C:\Users\mbucala\AppData\Local\Temp\MicrosoftEdgeDownloads\9f63b71f-41a4-4825-8568-160a000d76ca\11606_2023_8415_MOESM1_ESM.docx#_Toc143273413)

[Section F. Survey Results Tables 30](file:///C:\Users\mbucala\AppData\Local\Temp\MicrosoftEdgeDownloads\9f63b71f-41a4-4825-8568-160a000d76ca\11606_2023_8415_MOESM1_ESM.docx#_Toc143273416)

Table F.1. Comparative analysis of staff survey characteristics between intervention and control sites before and after implementation 30

[Table F.2. Comparative analysis of provider survey characteristics between intervention and control sites before and after implementation 31](file:///C:\Users\mbucala\AppData\Local\Temp\MicrosoftEdgeDownloads\9f63b71f-41a4-4825-8568-160a000d76ca\11606_2023_8415_MOESM1_ESM.docx#_Toc143273422)

[Table F.3. Responses to a question assessing agreement/disagreement with statements about portal messaging and in-basket workload. 32](file:///C:\Users\mbucala\AppData\Local\Temp\MicrosoftEdgeDownloads\9f63b71f-41a4-4825-8568-160a000d76ca\11606_2023_8415_MOESM1_ESM.docx#_Toc143273417)

[Table F.4. Frequency and reasons for dual routing of portal messages 33](file:///C:\Users\mbucala\AppData\Local\Temp\MicrosoftEdgeDownloads\9f63b71f-41a4-4825-8568-160a000d76ca\11606_2023_8415_MOESM1_ESM.docx#_Toc143273418)

[Table F.5. Factors impacting message routing and inbox prioritization practices 34](file:///C:\Users\mbucala\AppData\Local\Temp\MicrosoftEdgeDownloads\9f63b71f-41a4-4825-8568-160a000d76ca\11606_2023_8415_MOESM1_ESM.docx#_Toc143273419)

[Table F.6. Factors related to burnout and stress from portal messages 35](file:///C:\Users\mbucala\AppData\Local\Temp\MicrosoftEdgeDownloads\9f63b71f-41a4-4825-8568-160a000d76ca\11606_2023_8415_MOESM1_ESM.docx#_Toc143273420)

[Table F.7. Responses assessing agreement with statements about the impact of portal messaging on patient care and workflow 36](file:///C:\Users\mbucala\AppData\Local\Temp\MicrosoftEdgeDownloads\9f63b71f-41a4-4825-8568-160a000d76ca\11606_2023_8415_MOESM1_ESM.docx#_Toc143273423)

[Table F.8. Responses assessing workload factors related to portal messages and workflow, presented as means with standard deviations 37](file:///C:\Users\mbucala\AppData\Local\Temp\MicrosoftEdgeDownloads\9f63b71f-41a4-4825-8568-160a000d76ca\11606_2023_8415_MOESM1_ESM.docx#_Toc143273424)

[Table F.9. Percentage distribution of recommended response times for various types of patient portal messages 38](file:///C:\Users\mbucala\AppData\Local\Temp\MicrosoftEdgeDownloads\9f63b71f-41a4-4825-8568-160a000d76ca\11606_2023_8415_MOESM1_ESM.docx#_Toc143273425)

[Table F.10. Perceived barriers to timely handling of in-basket messages, presented as means with standard deviations 39](file:///C:\Users\mbucala\AppData\Local\Temp\MicrosoftEdgeDownloads\9f63b71f-41a4-4825-8568-160a000d76ca\11606_2023_8415_MOESM1_ESM.docx#_Toc143273426)

[Table F.11. Time consumption ratings for various in-basket message categories, presented as means with standard deviations 40](file:///C:\Users\mbucala\AppData\Local\Temp\MicrosoftEdgeDownloads\9f63b71f-41a4-4825-8568-160a000d76ca\11606_2023_8415_MOESM1_ESM.docx#_Toc143273427)

[Table F.12. Stress ratings for various in-basket message categories, presented as means with standard deviations. 41](file:///C:\Users\mbucala\AppData\Local\Temp\MicrosoftEdgeDownloads\9f63b71f-41a4-4825-8568-160a000d76ca\11606_2023_8415_MOESM1_ESM.docx#_Toc143273428)

[Section G. EHR Metrics Tables 42](file:///C:\Users\mbucala\AppData\Local\Temp\MicrosoftEdgeDownloads\9f63b71f-41a4-4825-8568-160a000d76ca\11606_2023_8415_MOESM1_ESM.docx#_Toc143273416)

[Table G.1. Absolute pre- and post differences for intervention and control sites, and differen-in-differences for various measures related to portal messaging 42](file:///C:\Users\mbucala\AppData\Local\Temp\MicrosoftEdgeDownloads\9f63b71f-41a4-4825-8568-160a000d76ca\11606_2023_8415_MOESM1_ESM.docx#_Toc143273416)

[Table G.2. Relative change for exploratory portal messaging 43](file:///C:\Users\mbucala\AppData\Local\Temp\MicrosoftEdgeDownloads\9f63b71f-41a4-4825-8568-160a000d76ca\11606_2023_8415_MOESM1_ESM.docx#_Toc143273416)

[Table G.3. Summary of denominator response rates 44](file:///C:\Users\mbucala\AppData\Local\Temp\MicrosoftEdgeDownloads\9f63b71f-41a4-4825-8568-160a000d76ca\11606_2023_8415_MOESM1_ESM.docx#_Toc143273416)

**Section A.** Timeline Highlighting Key Phases of the Project

The below project timeline showing the sequence of activities from July 2022 to September 2023. The key steps included problem analysis, deciding outcome measures, survey distribution, pre-intervention data collection, identification of pre-intervention findings, intervention development and implementation, the intervention phase, interviews to understand workflows, implementation of routing document and best practices feedback, and post-intervention survey distribution and data collection. The project also involved concurrent control sites (Site A and Site B).

**
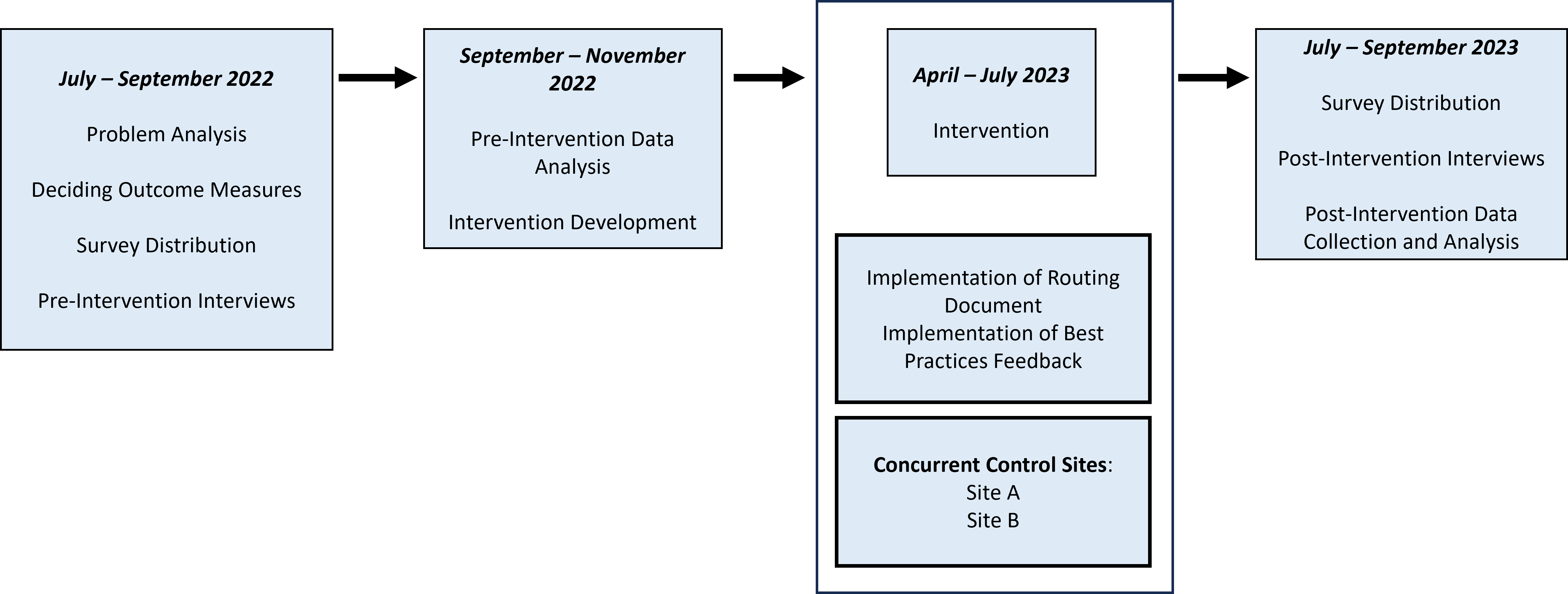
**

**Section B.** Pre-Intervention Interview Guide

Below are general questions for all types of clinic staff. Questions are open-ended. Some questions may be asked of one type of staff member, and not another. Follow-up questions will be asked based on what responses the interviewee provides. Some possible probing questions are listed under the main questions.

**Instructions to begin the interview:**

- Introduce team (whoever is present)
- Review the purpose of the project with each participant; make sure they understand and answer any questions.
  1. *Adoption of electronic health records has led to increased electronic communication amongst healthcare providers and patients through portal messages. The amount of time clinic staff and health care providers spend managing portal messages has sky-rocketed during the pandemic, leading to patient care delays, stress and burnout. For this project we are interested in learning more about the current standard workflow used to manage portal messages. The goal is to understand the current processes and priorities of staff and identify processes that are working well and processes that could improve. We are interested in your ideas so we can work to develop and test out new strategies.*
- Guarantee confidentiality - you will not be identified by name in any reports or other publications
- Remind participants that they can choose not to answer a question and/or stop whenever they would like
- Ask for permission to record the interview so we do not miss anything they say

**All Staff Questions (icebreaker / warm up):**

1. What clinic do you work at?
2. Can you please describe your position at the clinic?

*Probes:*

- 1. How many years have you been in this position?

1. How many years have you been in the healthcare field?

**[Portal Messages: General]**

1. What are some of the uses to the patient portal that you think are most useful?
2. What tasks or types of issues does the portal work best to accomplish?
3. What types of messages do you wish were communicated in a different medium than the portal?
4. What changes have you noticed with portal messages since the pandemic?

*Probes:*

- 1. Could you give an example?
  2. Volume of messages
  3. Time spent answering messages
  4. Complexity of message

**Questions for PCPs only:**

**[Current Workflow: Triage of Portal Messages]**

1. What is your understanding of triage process for portal messages at your clinic? For example, what happens to portal messages before they come to your in-box?
2. Would you find a standardized triage protocol for portal messages in the clinic to be useful?
   1. Why or why not?
   2. If yes, are there portal messages that come to your in-box that you think could be managed by other members of the care team? Could you give some examples?
   3. What types of messages is it important for you to handle?
3. Are there portal messages that come to your inbox that warrant a patient visit (either office or virtual)?
   1. What were the reasons for this?
   2. What types of portal messages do you think should automatically be flagged as needing a patient visit?

**[Current Workflow: Pre-Work around Portal Messages]**

1. Are there times when other members of the clinic do some “pre-work” around the portal message before they come to your in-box?
   1. If yes, could you describe this process?
   2. How well does it work?
   3. What types of messages does it work best for?
   4. Are there types of messages that you think more “pre-work” could be done for?

**[Current Workflow: Communication Quality around Portal Messages]**

1. After other members of the care team do “pre-work” around portal messages and forward the message to you, do they normally include a note to you with the message?
   1. If so, could you talk about the quality of this communication?
   2. When do these notes work well?
   3. In what situations could they be improved?

**Questions for other types of staff who triage messages (e.g., MAs, Nurses, LPNs, Clerks):**

**[Current Workflow: Triage of Portal Messages]**

1. Could you walk us through the process for handling portal messages at your clinic?
   1. For example, when a patient enters a message into the portal, what happens to it?
   2. Who typically handles the message first?
   3. How is it decided who will handle the message first?
   4. When to you review portal messages within your day?
2. Do you receive any training on how to answer or triage portal messages?
   1. Have you received training or instructions on what messages need to be forwarded to the PCP?
   2. Are there messages that are never sent to the PCP?
   3. How do you decide?
3. How do you decide what messages get forwarded to the Primary Care Physician vs what messages are handled by you or other clinic staff?
4. When does this process work well?
5. When does this process not work well?
6. What are the most common reasons you forward messages to the PCP?
   1. For example, is it messages that out of your clinical scope?
   2. Or do you regularly forward messages because you are not trained to answer them, such as addressing orders needed?
   3. Is it questions linked to prior PCP visits that the PCP would want to answer?
7. Would you find a standardized triage protocol for portal messages in the clinic to be useful?
   1. Why or why not?
   2. If yes, what would this protocol look like?

**[Current Workflow: Pre-Work around Portal Messages]**

1. Are there times when you do some “pre-work” around the portal message before sending it to the PCP?
   1. If yes, could you describe this process?
   2. How does it work?
   3. What types of messages does it work best for?

**[Current Workflow: Communication Quality around Portal Messages]**

1. After you do “pre-work” around portal messages and forward the message to the PCP, do you normally include a note with the message?
   1. If so, could you talk about how you decide what to include with the note?
   2. When do these notes work well?

**Questions for all types of interviewees:**

**[Managing Patient Expectations]**

1. How quickly do your patients expect that you will answer their portal messages?
2. What do you think is a reasonable amount of time for turnaround response on patient messages?
3. Would you find patient education on how to use the portal to be useful?
   1. Why or why not?
   2. If yes, what should it include?

**[Stress and Burnout]**

1. Now let’s talk about stress and burnout. What types of messages seem to take the most time to resolve, or cause the most stress?
2. What barriers hinder your ability to answer a portal message within 24-48 hours?
3. What would relieve some of your stress and burnout?

**Wrap-up**

1. Do you have any other suggestions for ways to improve portal management in primary care?
2. Is there anything else that you would you like to tell us?

That is the end of the questions that we have for you. Now do you have any questions for me or any other comments on anything that we have discussed today?

Thank you very much for taking the time to participate in this interview. We greatly appreciate you making the effort as we know your time is very valuable.

**Section B.** Post-Intervention Interview Guide

This is an interview guide targeting all types of clinical staff. Questions are open-ended. Follow-up questions will be asked based on what responses the interviewee provides. Some possible probing questions are listed under the main questions.

Instructions to begin the interview:

- Introduce team (whoever is present)
- Review the purpose of the project with each participant; make sure they understand and answer any questions.
  1. Portal message use has sky-rocketed since the pandemic, as a result the Division of General Medicine has funded a project in [clinic name redacted] to work on improving portal messaging, led by [investigator name redacted]. This project has two parts that began on April 24th, (1) best practices for routing portal messages were shared with staff and physicians, (2) instituted a half-day a week of dedicated time for medical assistants to triage portal messages to providers. We are interested in your thoughts on these tools and dedicated staff time. As a reminder, during this interview, we are only focusing on patient portal messaging, and will not be focusing on any other MiChart or call center messages.
- Guarantee confidentiality - you won’t be identified by name in any reports or other publications
- Remind participants that they can choose not to answer a question and/or stop whenever they would like
- There are no right or wrong answers. You are the experts, and we want to learn from you.
- Make participants aware that there is a link to an anonymous Qualtrics survey located in the chat during the entirety of the meeting. We encourage you to utilize the survey to provide us with any feedback that we did not have a chance to cover during the meeting.
  1. Link to the survey: (https://umichumhs.qualtrics.com/jfe/form/SV_5ojdhTqhNjkVAR8)
- Ask for permission to record the group interview so we don’t miss anything they say
- At this point in the interview, the “in-basket messaging best practices” tool should be displayed on the screen for the participants to see.

**GROUP INTERVIEW**

**Feedback of in-basket messaging interventions**

1. To start, could everyone please introduce yourself by letting us know what your role is and how long you’ve worked at [clinic name redacted]?
2. Have you seen the “portal messaging best practice” information document or the routing document that are printed out here prior to today?
   1. If so, where have you seen them?
3. What are your general impressions of the “portal messaging best practice” information and routing documents?
   1. What aspects worked well in the clinic?
   2. What aspects did not work well?
   3. Anything you would recommend changing or improving for the information provided?
4. Now let’s talk a little more about the “portal messaging best practices” tool. Did you find this to be useful?
   1. Why or why not?
   2. If yes, when did you reference the document?
   3. If yes, approximately how often did you reference the tool?
   4. If yes, how did you access the document?
   5. Did you share the document with anyone else?
   6. Is there anything you think the tool is missing?
5. Next, let’s look a little closer at the routing document. Did you find the routing document to be useful?
   1. Why or why not?
   2. If yes, when did you reference the document?
   3. If yes, approximately how often did you reference the tool?
   4. If yes, how did you access the document?
   5. Did you share the document with anyone else?
   6. Is there anything you think the tool is missing?
6. Not including residents, did you notice any changes to the way in-basket messaging was conducted following the rollout of these two documents, which started at the end of April?
   1. If so, please describe.
7. We are aware the [clinic name redacted] clinic was particularly short-staffed in the month of May when we only had 1 LPN managing all portal messages without an embedded RN assisting. Did this have any impact on portal work you performed?
   1. Could you provide an example?
   2. Did the short staffing have any impact on your ability to follow the portal messaging best practices?
8. As a reminder, for this project we also instituted a half-day a week of dedicated time for 1-2 medical assistants to triage portal messages to providers. Did you find the dedicated time for medical assistants to help triage portal messages useful?
   1. For RNs ONLY: Did it make a meaningful difference in reducing the typical high volume of Mondays?
   2. For RNs and MAs ONLY: What were your thoughts on the length of time – one half day – that the dedicated MA had to do this?
   3. For MDs and MAs ONLY: Did having a medical assistant pulled from clinical duties for this half day have any effect on clinic flow?

**Sustainability & Ideas for Improvement**

1. Would you like to see the “ portal messaging best practices” and routing document continue at [clinic name redacted], if so, how would like it to be emphasized e.g., email pushes vs huddles, or anything else?
   1. If so, what would be the best way to provide that information (i.e., huddles)?
2. Would you like to see the dedicated time for MAs for portal message management continued?

**Stress and Burnout**

1. Now let’s talk about stress and burnout related to portal messaging. Since the pilot launched in April, have you noticed any changes since the launch in the time it has taken to resolve messages?
   1. If so, please describe.
   2. If so, has this had any effect on your stress and/or burnout?

**Future Considerations - For MDs ONLY:**

1. Due to the large number of physicians at [clinic name redacted] (including residents) and variable time spent in clinic, one challenge we faced was disseminating information and re-enforcing portal best practices for our physicians. What suggestions do you have for how this might be done in future projects?
2. What are your thoughts implementing the role of an “inbox ologist” to serve as a designated staff member tasked with managing in-basket messaging while not impacting continuity of patient care?

**Wrap-up**

1. Do you have any other suggestions for ways to improve in-box management in primary care?
2. Is there anything else that you would you like to tell us?

That is the end of the questions that we have for you. Now do you have any questions for me or any other comments on anything that we have discussed today?

Thank you very much for taking the time to participate in this interview. We greatly appreciate you making the effort as we know your time is very valuable

**Section C**. Portal Messaging Best Practice Standards

| **Recommended Best Practice Standards** | **PSA** | **MA** | **LPN** | **RN** | **MD/APP** |
| --- | --- | --- | --- | --- | --- |
| **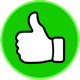 START** |  |  |  |  |  |
| 1. Direct **all replies to a pool** rather than an individual person. For example:    1. All CPAP issues or patient requests for orders should be routed to the MA pool only    2. All patient queries about medication authorizations should be routed to the MA pool only | O | O | O | O | O |
| 1. All **new symptom** complaints should be routed to RN pool for further triage | O | O | O |  |  |
| 1. New symptom complaints should prompt a phone call for further triage    1. If two call attempts are made with failure to connect with patient, route to provider 2. Judgment can be applied to recommend a visit for triaged new symptom complaints and forwarded to admin pool without involving the provider |  |  |  | O |  |
| 1. When replying directly to portal messages with specific queries to the patient, check “Reply to me” whenever possible and appropriate |  |  |  |  | O |
| **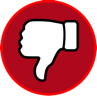 STOP** |  |  |  |  |  |
| 1. **Avoid** routing to more than one pool at a time, or to individual + pool | O | O | O | O | O |
| 1. **Avoid** “FYI” messages or forwarding threads that do not require any action from pool or provider | O | O | O | O | O |
| 1. **Avoid** “Please advise” or “Next steps” replies and instead suggest a possible plan of care where appropriate |  | O | O | O |  |

**Section C**. Staff Routing Guide

MICHART TIP: You do not have to manually type in these pools for routing each time. You can save various pools under a dropdown of “My List” for routing call encounters, portal messages and results. Hit the dropdown, select “Build my lists” and then add the various pools we use. If unsure how, can ask [name redacted or our clinic’s MiChart superuser, [name redacted]

P TC CARE NAVIGATOR CLIN SUP

- Patients you wish to refer for care navigator support
  - Care navigation follows patients with active medical issues who need help navigating the health care system
  - The ideal referral is patients with 2 or more chronic health conditions (e.g., CHF, COPD, cancer, diabetes, cognitive impairment) whose treatment of these conditions is limited by some psychosocial factor (e.g., low literacy, missing frequent appointments, limitations in mobility and self-care)
  - Patients may also be referred as part of transition of care after discharge for patients who have had complicated hospital stays with anticipated difficulties with care coordination post-discharge
  - If unsure of appropriateness, may discuss with RN CN [name redacted]

P TC GEN MED ADMIN

- Schedule requests, from provider or patient
- Scheduling issues, conflicts, or errors
- Converting visits to face-to-face, video or phone
- Reverse appointment check-in
- Assistance locating a contact number for a specialty clinic, radiology, or diagnostic unit
- Obtaining a “Release of Information” for outside records
  - Note: Mental health records requests require a separate, special form
- Accepting outside records to be scanned into chart

P TC GEN MED BILLING

- For all patient billing-related questions, including visit billing codes and insurance questions
- May also help coordinate peer-to-peer consultations

P TC GEN MED LPN

- Prescription refill requests and issues
- Verbal ok for home care (including nurse home care, PT and OT)
- Communication of normal results to patients
  - Whenever possible, relay results over portal rather than involving LPN
- Call to relay messages on behalf of physicians to pharmacies, staff within the healthcare system, or other entities who call in asking for input from the clinic

P TC GEN MED MA

- Medication prior authorizations
- Forms and anything related to provider folders and faxes
- Faxing documents (e.g., forms, external referrals, or external orders)
- Inputting external vaccine records
- Durable medical equipment orders
  - CPAP orders
    - There needs to be a visit or telephone note documenting that the patient was informed they have OSA, that PAP therapy nightly was recommended as the first line, and patient is aware that treatment of OSA has important health benefits, including blood pressure, sleep quality, and for overall cardiopulmonary health
      - Can use dot phrase from [name redacted] – “NHOSA”
      - A nurse call note relaying results is not enough. However, physicians do not need to call patient themselves even if not discussed in a visit. Once nurse calls patient, physicians can simply write a separate telephone note documenting that the patient was informed of sleep study results and need for PAP therapy, as well as the importance of adherence in treatment
  - Diabetes testing supplies orders
    - Visit note must document frequency of testing
  - Urinary catheter orders
  - Incontinence supplies
    - Covered for patients with Medicaid plans, and some limited Blue Cross plans
    - Orders must be signed by an attending
  - Nebulizers
  - Supplemental oxygen orders
    - Qualify if SpO2 88% at rest on room air, or desats with exertion
    - No other staff in our clinic but physicians can hall walk patients to qualify for supplemental oxygen for hypoxia with exertion. For most physicians, it will be unrealistic to perform protocol during a clinic day
    - Instead, can have this done at PFT lab. Order “PFT Adult Walk tests” and select “PFT Adult Oxygen assessment with ambulation”
  - Wheelchair orders
    - Must document in a note the medical necessity for use of a wheelchair within the home. Insurance will not cover wheelchairs for use outside the home
    - If this is a power wheelchair, must also document patient is unable to propel themselves in a manual wheelchair
  - Compression stockings and orthotics (if external)
    - Internal orders are fulfilled at UM Orthotics on Industrial Drive; patient needs to call 734-973-2400
    - For external orders, patient needs to contact insurer to find out which DME company they can fulfill order in their area. We can hand them printed order to take with them there, or they can give us number to fax to the location they will use

P TC GEN MED REFERRAL

- Obtaining more information related to referral requests from patients
- Obtaining global authorization on referrals
- Assist in prior authorizations for referrals
- Assistance backdating referrals for patients

P TC GEN MED RN

- Communication of abnormal results to patients
- Symptom triage
- Phone follow up on clinical status following prior patient contact with the clinic
- Patient education on certain common topics, e.g., self-administration of new subcutaneous injections

CLINIC SOCIAL WORKER – No pool, current social worker is [name redacted]

- Psychotherapy referrals
- Mental health care coordination, e.g., external psychiatry, ADHD testing and other neuropsychic testing
- Substance/alcohol use concerns or treatment*
- Suicidality and safety concerns
- Neglect or intimate partner violence
- Legal guardianship questions
- Advanced care planning support and DPOA paperwork
- Insurance and disability questions

*For opiate use disorder, there is additional support through “MAT RN”
 Can directly message [name redacted] or make referral using “Primary Care Buprenorphine”
 SMARTSET (under SMARTSETS, not regular order entry)

GUEST ASSISTANCE PROGRAM (GAP) REFERRAL – Current clinic contact is [name redacted]

- Transportation issues
- Food insecurity and financial concerns
- Prescription costs
- Legal concerns
- Employment resources
- Potentially covering medical equipment and supplies (e.g., BP machine)

BEHAVIORAL HEALTH COLLABORATIVE CARE (BHCC) – Current BHCC SW [name redacted]

- Referrals for patients with fairly straightforward anxiety and depression
- Assist with assessment and assistance with medication titration
- Can assist with community referrals for therapy for patients enrolled with them

MISCELLANEOUS – For physicians, with assistance from RNs and RN CN

- Home care orders (e.g., home PT, OT, RN)
  - Verbal oks are allowable for home care orders following inpatient stays, and are routed to LPNs
  - Home care orders done without a hospitalization:
    - Require an outpatient visit (face-to-face or video, NO PHONE) documenting in note the reasons patient needs home care
    - No current pathway for order to be queued for physician. Physician must place and sign order
    - Type “In Home Care Order” and select the services, go through all hard-stops and “F2” through the comments, answering all questions
    - Select “Outpatient Case Management” for “Who will arrange” question; this ensures this will be managed once order is signed, and no other steps are needed from the clinic to follow through
- The clinic is working out a process for the following, less common orders. But physicians should note the following:
  - Hospital bed
    - Must have in-person or video visit documenting need for bed
    - Covered indications are for cardiac, pulmonary or pain reasons only. Will not be covered for safety, fractures, inability to climb stairs to bedroom, etc.
  - Shower bench
    - Only covered if they have Medicaid only. Medicaid + Medicare would not work
    - If no Medicaid and finances tight, place GAP referral

**Section D.** Pre- and Post-Intervention Staff Survey

Consent We are studying the in-basket messaging and how the triage system for portal messaging works across clinics to find ways to better the system and reduce stress and burnout related to portal messaging. This survey will be used to gain insight about current portal messaging triage practices and experiences. Please give consent to participate in this survey.

- I consent
- I do not consent

1. Please select your clinic

- [Clinic name redacted]
- [Clinic name redacted]
- [Clinic name redacted]
- [Clinic name redacted]

1. Please select your role

- RN
- LPN
- MA
- Patient Services/Clinic Staff

1. How many years have you held this role?

- 0-2 years
- 3-5 years
- 6-10 years
- 11-20 years
- More than 20 years

1. How many years have you been in your role at your specific clinic site?

- 0-2 years
- 3-5 years
- 6-10 years
- 11-20 years
- More than 20 years

1. The following statements are about portal messaging and in-basket workload. Please indicate your level of agreement related to each statement.

|  | Strongly Agree | Somewhat Agree | Neither Agree nor Disagree | Somewhat Disagree | Strongly Disagree | N/A or Don't Know |
| --- | --- | --- | --- | --- | --- | --- |
| Use of portal messages with patients has a positive impact on patient care |  |  |  |  |  |  |
| Use of the portal and portal messages has a positive impact on patient satisfaction |  |  |  |  |  |  |
| Patients expect the majority of messages to be seen and handled by their PCP |  |  |  |  |  |  |
| The expectations of all clinical team member roles in responding to portal messages is clear |  |  |  |  |  |  |
| My clinic has clear, standardized guidelines on how to address various portal messages received |  |  |  |  |  |  |
| I received adequate training and guidance on how to manage portal messages efficiently and confidently |  |  |  |  |  |  |
| There is high variability in how involved providers want to be with portal messages (especially if the PCP is a resident) |  |  |  |  |  |  |

|  |  |
| --- | --- |

1. The following statements are about portal messaging and in-basket workload. Please indicate your level of agreement related to each statement.

|  | Strongly Agree | Somewhat Agree | Neither Agree nor Disagree | Somewhat Disagree | | Strongly Disagree | N/A or Don't Know | |
| --- | --- | --- | --- | --- | --- | --- | --- | --- |
| There is high variability in how providers want types of portal messages handled |  |  |  |  |  | |  |  |
| Portal messages routed back to my box as an FYI (with no further action needed from me) is appropriate and expected |  |  |  |  |  | |  |  |
| Portal messages routed back to a provider or another pool as an FYI (with no further action needed from them) is appropriate and expected |  |  |  |  |  | |  |  |
| When portal messages are routed to my pool’s box and another pool or person, it is less clear to me where the responsibility of the message lies |  |  |  |  |  | |  |  |
| At my clinic’s current level of staffing, I feel I have the bandwidth and time to address portal messages completely and to the top of my license |  |  |  |  |  | |  |  |
| When the clinic is short-staffed I do not have time to complete the chart review or pre-work that I would like to before routing messages |  |  |  |  |  | |  |  |
| I can answer all portal messages routed to my team’s in-box during a normal 40-hour work week (if a 1.0 FTE) |  |  |  |  |  | |  |  |

*:*

1. (For Registered Nurses Only) The following statements are about portal messaging and in-basket workload. Please indicate your level of agreement related to each statement.

|  | Strongly Agree | Somewhat Agree | Agree | Neither Agree nor Disagree | Disagree | | Somewhat Disagree | Strongly Disagree | N/A or Don't Know | |
| --- | --- | --- | --- | --- | --- | --- | --- | --- | --- | --- |
| When I am routed symptom-related portal messages, I often answer the patient and collect information via a phone call |  |  |  |  | |  |  |  | |  |
| When I am routed symptom-related portal messages, I often answer the patient and collect information via a portal message back to the patient |  |  |  |  | |  |  |  | |  |

1. Frequency The following statements are about portal messaging and in-basket workload. Please indicate the frequency in which you typically encounter these situations.

|  | Several Times a Week | 1-2 Times a Week | Less Than Once a Week | 1-2 Times a Month | Less Than Once a Month | Never | N/A or Don't Know |
| --- | --- | --- | --- | --- | --- | --- | --- |
| I route portal message encounters to multiple people at once (e.g. RN pool and provider, or an individual staff member and team pool) |  |  |  |  |  |  |  |
| When portal messages seem urgent, I will route it to multiple providers and/or pools to ensure it is handled quickly |  |  |  |  |  |  |  |
| When there are too many portal messages in the in-basket competing with other work demands, I route them onwards to be addressed |  |  |  |  |  |  |  |
| Portal messaging interrupts my in-office clinic duties and patient care |  |  |  |  |  |  |  |

1. The following statements are about portal message volumes and in-basket workload. Please indicate your level of agreement related to each statement.

|  | Strongly agree | Agree | Somewhat Agree | Neither Agree nor Disagree | Somewhat Disagree | Disagree | Strongly Disagree | N/A or Don't Know |
| --- | --- | --- | --- | --- | --- | --- | --- | --- |
| Longer wait times when patients call in impact portal message volumes |  |  |  |  |  |  |  |  |
| There is a difference in portal message volume pre-COVID compared to present |  |  |  |  |  |  |  |  |
| There is a difference in portal message volume prior the Centralized Nurse Triage compared to present |  |  |  |  |  |  |  |  |
| Portal message volume varies by day of the week |  |  |  |  |  |  |  |  |
| I frequently receive duplicate encounters in multiple areas of the in-basket for the same patient issue |  |  |  |  |  |  |  |  |

1. The following question asks about your perception of portal messaging volume before and during the COVID-19 pandemic.

|  | Strongly agree | Agree | Somewhat Agree | Neither Agree nor Disagree | Somewhat Disagree | Disagree | Strongly Disagree | N/A or Don't Know |
| --- | --- | --- | --- | --- | --- | --- | --- | --- |
| COVID Compared to pre-COVID, the volume of portal messages has |  |  |  |  |  |  |  |  |
| Increased  Decreased |  |  |  |  |  |  |  |  |

1. The following question asks about your perception of portal messaging volume before the Central Nurse Triage was implemented.

|  | Strongly agree | Agree | Somewhat Agree | Neither Agree nor Disagree | Somewhat Disagree | Disagree | Strongly Disagree | N/A or Don't Know |
| --- | --- | --- | --- | --- | --- | --- | --- | --- |
| CNT Compared to prior to the Central Nurse Triage, the volume of portal messages has |  |  |  |  |  |  |  |  |
| Increased  Decreased |  |  |  |  |  |  |  |  |

1. What days of the week is portal message volume so high that it impacts speed of response and/or thoroughness in addressing the message before routing (select all that apply)

- Monday
- Tuesday
- Wednesday
- Thursday
- Friday
- The day of the week does not impact my ability to address portal messages quickly and completely

1. Think about the typical portal messages you get within your workday. What do you think is a feasible amount of time to turnaround a response (given current staffing levels and message volumes), from the clinic to a patient, after the patient sends a portal message?

- Less than 24 hours
- 24-48 hours
- 49-72 hours
- Within 1 week

1. Think about the typical portal messages you get within your workday. What do you think is an ideal amount of time to turnaround a response, from the clinic to a patient, after the patient sends a portal message?

- Less than 24 hours
- 24-48 hours
- 49-72 hours
- Within 1 week

1. I route messages to both a provider and pool for the following reasons.
   Please rank in order of frequency, from most common to least common:

______ Urgent Message

______ Unsure who can handle the message best

______ Provider should be included on most messages to ensure there is nothing else to add

1. Limit Ability What types of things limit your ability to respond to portal messages within 48 hours? Please rank from most important/frequent to least important/least frequent:

______ In-clinic patient care duties

______ Not enough information in initially routed message

______ Too many messages in one day

______ Messages are not routed to me same day

______ Clerical work like faxes and forms

______ Issue is not appropriate for the portal

______ Unsure how to accomplish the task in the EMR (Technical knowledge gap)

______ Ambiguous routing (multiple providers/pools included, unclear if it was my responsibility to manage)

1. Prioritize How do you prioritize your in-basket work?
   Please rank from highest priority to lowest priority.

______ Call Encounters

______ Medication Refills

______ Portal Messages

______ Result Messages from Providers

______ Faxes/Forms

______ Message Non-Encounters

1. The following questions touch on work-life balance

|  | Strongly Agree | Somewhat Agree | Neither Agree nor Disagree | Somewhat Disagree | Strongly Disagree | N/A or Don't Know |
| --- | --- | --- | --- | --- | --- | --- |
| Clerical burden contributes to my feelings of burnout or stress |  |  |  |  |  |  |
| Portal messages contribute to my feelings of burnout or stress |  |  |  |  |  |  |
| Current staffing shortages contribute to my feelings of burnout or stress |  |  |  |  |  |  |

1. Rank Stress What factors about portal messages contribute to your stress and burnout?

   Please rank from the highest contributing factor to lowest contributing factor

______ Patient expectations in speed of response (1)

______ Patient expectations for what can be managed on the portal (2)

______ Confusion generated by multiple encounters/messages for the same patient issue (3)

______ Variability in provider expectations about portal message handling (4)

______ Provider communication responses for portal handling (5)

______ Delays in providers addressing portal messages once routed to them (6)

______ Lack of training or protocols to guide me on how to manage certain messages expected of me (7)

______ Balancing addressing portal messages while engaged in in-clinic duties (8)

1. What aspects of portal messaging are barriers to efficient and high-quality workflow and patient care?

________________________________________________________________

1. What aspects of portal messaging facilitate efficient and high-quality workflow and patient care?

________________________________________________________________

1. What are 1-2 things you would like to improve about portal messaging workflow? Consider aspects within the control of the clinic i.e., not what a patient messages about, or how a patient responds.

________________________________________________________________

**Section D.** Pre- and Post-Intervention Provider Survey

We are studying the in-basket messaging and how the triage system for portal messaging works across clinics to find ways to better the system and reduce stress and burnout related to portal messaging. This survey will be used to gain insight about current portal messaging triage practices and experiences. 
Please give consent to participate in this survey.

- I consent
- I do not consent

1. Please select the clinic you work in

- [Clinic name redacted]
- [Clinic name redacted]
- [Clinic name redacted]
- [Clinic name redacted]

1. Please select your role at your clinic

- Physician
- APP
- Resident

1. How many years have you been practicing?

- 0-2 years
- 3-5 years
- 6-10 years
- 11-20 years
- More than 20 years

1. How many years have you been practicing at your current clinic?

- 0-2 years
- 3-5 years
- 6-10 years
- 11-20 years
- More than 20 years

1. The following statements are about portal messaging and in - basket workload. Please indicate your level of agreement related to each statement.

    *Here, working hours are defined as both clinical hours and administrative hours, such as 1.0 FTE working 32 clinical hours and 8 administrative hours (that may not necessarily be taken during the typical 8am-5pm M-F timeframe).*

|  | Strongly Agree | Agree | Somewhat Agree | Neither Agree nor Disagree | Somewhat Disagree | Disagree | | Strongly Disagree | N/A or Don't Know | |  |
| --- | --- | --- | --- | --- | --- | --- | --- | --- | --- | --- | --- |
| Use of portal messages with patients has a positive impact on patient care |  |  |  |  |  |  |  | | |  | |
| Use of the portal and portal messages has a positive impact on patient satisfaction |  |  |  |  |  |  |  | | |  | |
| The expectations of all clinical team member roles in responding to portal messages is clear |  |  |  |  |  |  |  | | |  | |
| I can answer all of my portal messages during my standard working hours (e.g., 40 hours per 1.0 FTE) |  |  |  |  |  |  |  | | |  | |
| The majority of portal messages I am routed require physician input another staff member cannot address |  |  |  |  |  |  |  | | |  | |
| The majority of portal messages I am routed contain enough info that I can successfully manage them without much back and forth with patients |  |  |  |  |  |  |  | | |  | |

1. How much time outside of your standard working hours do you spend addressing portal messages per week?

- No, I complete all portal messaging work within my standard working hours
- 1-2 hours
- 3-4 hours
- 5-6 hours
- 7 or more hours

1. When a portal message is sent following up on a question about a visit or diagnostics done by a non-PCP physician or APP of the practice it should be routed to:

- PCP only
- Non-PCP physician or APP who saw the patient
- Both PCP and non-PCP physician or APP who saw the patient
- Other

1. These questions focus on your portal message usage.

|  | Daily | 1-2 times a week | | | Less than once a week | 1-2 times a month | | Less than once a month | Never | | N/A or don't know |
| --- | --- | --- | --- | --- | --- | --- | --- | --- | --- | --- | --- |
| I manage patient care issues via portal messaging that would have otherwise required an office visit |  | |  |  | |  |  | | |  |  |
| I manage patient care issues via portal messaging that should be converted into an office visit |  | |  |  | |  |  | | |  |  |
| I manage patient care issues via portal messaging that would have otherwise been a nurse phone call |  | |  |  | |  |  | | |  |  |
| I manage patient care issues via portal messaging that should be converted to a nurse phone call |  | |  |  | |  |  | | |  |  |
| I route portal messages back to staff requesting more information |  | |  |  | |  |  | | |  |  |
| Portal messaging interrupts my in-clinic duties and patient care |  | |  |  | |  |  | | |  |  |

1. Think about the typical portal messages you get within your workday. What do you think is a feasible amount of time to turnaround a response (given current staffing levels and message volume) from the clinic to a patient after the patient has sent a portal message?

- Less than 24 hours
- 24-48 hours
- 49-72 hours
- Within 1 week

1. Think about the typical portal messages you receive within your workday. What do you think is an ideal amount of time to turnaround a response from the clinic to the patient after the patient sends a portal message?

- Less than 24 hours
- 24-48 hours
- 49-72 hours
- Within 1 week

1. What types of things limit your ability to respond to portal messages within 48 hours? Please rank from most important/frequent to least important/least frequent:

______ Seeing patients that day

______ Not enough information in initially routed message

______ Too many messages in one day

______ Messages are not routed to me same day

______ Issue is not appropriate for the portal

______ Unsure how to accomplish the task in EMR (technical knowledge gap

______ Ambiguous routing (multiple providers/pools included, unclear if it is my responsibility to manage)

1. Thinking of the last month, how often are the following pended appropriately with the necessary information before they are routed to you?

|  | 0% - 25% | 26% - 50% | 51% - 75% | 76% - 100% |
| --- | --- | --- | --- | --- |
| Orders |  |  |  |  |
| Prescription Refills |  |  |  |  |
| Patient Portal Messages |  |  |  |  |
| Telephone Encounters |  |  |  |  |

1. Please indicate your level of agreement with the following questions that touch on work-life balance

|  | Strongly disagree | Disagree | Somewhat disagree | Neither agree nor disagree | Somewhat agree | Agree | | Strongly agree | | N/A |
| --- | --- | --- | --- | --- | --- | --- | --- | --- | --- | --- |
| Clerical burden contributes to my feelings of burnout and stress |  |  |  |  |  | |  | |  |  |
| Portal messages contribute to my feelings of burnout and stress |  |  |  |  |  | |  | |  |  |

1. What types of patient portal messages take the most time (as currently handled)? Please rank from the most time consuming to the least time consuming :

______ Medication Refill Requests (1)

______ Medication Questions (2)

______ Post Visit Follow Up (3)

______ Questions About New Symptoms (4)

______ Referral Requests (5)

______ Personal Updates (6)

______ Mental Health Concerns (7)

______ Schedule Requests (8)

______ Administrative Issues and Forms (ex: FMLA) (9)

1. What types of patient portal messages cause the most stress/frustration? Please from the most stressful to the least stressful:

______ Medication Refill Requests (1)

______ Medication Questions (2)

______ Post Visit Follow Up (3)

______ Questions About New Symptoms (4)

______ Referral Requests (5)

______ Personal Updates (6)

______ Mental Health Concerns (7)

______ Schedule Requests (8)

______ Administrative Issues and Forms (ex: FMLA) (9)

1. How quickly should the following types of patient portal messages be answered (within business days and hours : 7am - 5pm , Monday - Friday ) ?

|  | Before the end of the business day | Within 24 hours | 24-48 hours | | 49-72 hours | Within 1 week |
| --- | --- | --- | --- | --- | --- | --- |
| Medication Refill Requests |  |  |  |  | |  |
| Medication Questions |  |  |  |  | |  |
| Post Visit Follow Up |  |  |  |  | |  |
| Questions About New Symptoms |  |  |  |  | |  |
| Referral Requests |  |  |  |  | |  |
| Personal Updates |  |  |  |  | |  |
| Mental Health Concerns |  |  |  |  | |  |
| Schedule Requests |  |  |  |  | |  |
| Administrative Issues and Forms (ex: FMLA) |  |  |  |  | |  |

1. What aspects of portal messaging are barriers to an efficient and high-quality workflow and for patient care?

________________________________________________________________

1. What aspects of portal messaging facilitate an efficient and high-quality workflow and for patient care?

________________________________________________________________

1. Have you ever had an incident of a near-miss or a poor outcome because of a concerning clinical problem that was routed in a portal message and not addressed within the timeframe you would have liked? If yes, please describe.

________________________________________________________________

1. What are 1 - 2 things you would like to improve about the portal messaging workflow? Consider aspects within the control of the clinic , i.e., not what a patient messages about, or how a patient responds

________________________________________________________________

**Table E.1.** Definitions for portal messaging EHR metrics.

| **EHR Metric** | **Description** |
| --- | --- |
| Messages to Physicians | Total number of portal messages received by physicians relative to physician cFTE |
| Messages Sent by  Physicians | Total number of portal messages sent by physicians relative to physician cFTE |
| Encounters with Physician First Non-Patient Sender | Total number of portal messages where the physician was the first non-patient sender in an encounter per 1000 messages |
| Carbon Copy Messages | Total number of portal messages where multiple recipients received the same message simultaneously per 1000 messages |

**Table F.1.** Comparative analysis of staff survey characteristics between intervention site and control sites before and after implementation

|  | **Intervention Site** | | **Control Sites** | |  |  |
| --- | --- | --- | --- | --- | --- | --- |
|  | | | **Pre** | **Post** | **Pre** | **Post** |
| Staff survey characteristics | | | | | |  |
| Total, n | | 8 | 11 | 29 | 30 |  |
| RN, n (%) | | 1 (12%) | 3 (27%) | 8 (26%) | 7 (23%) |  |
| LPN, n (%) | |  | 1 (9%) | 2 (6%) | 5 (17%) |  |
| MA, n (%) | | 4 (50%) | 6 (55%) | 8 (26%) | 6 (20%) |  |
| PSA, n (%) | | 3 (38%) | 1 (9%) | 11 (40%) | 12 (40%) |  |
| Time in role | | | | | |  |
| 0-2 years, n (%) | | 2 (25%) | 1 (9%) | 5 (17%) | 7 (23%) |  |
| 3-5 years, n (%) | |  | 2 (18%) | 11 (37%) | 7 (23%) |  |
| 6-10 years, n (%) | | 3 (38%) | 1 (9%) | 3 (10%) | 8 (28%) |  |
| 11-20, n years (%) | | 2 (25%) | 4 (37%) | 7 (24%) | 7 (23%) |  |
| More than 20 years, n (%) | | 1 (12%) | 3 (27%) | 10 (3%) | 1 (3%) |  |
| Time at clinic | | | | | |  |
| N/A | |  | 1 (9%) |  |  |  |
| 0-2 years, n (%) | | 4 (50%) | 3 (27%) | 13 (45%) | 12 (40%) |  |
| 3-5 years, n (%) | | 1 (12)% | 1 (9%) | 13 (45%) | 13 (43%) |  |
| 6-10 years, n (%) | | 1 (12%) | 2 (18%) |  | 2 (7%) |  |
| 11-20 years, n (%) | | 2 (26%) | 4 (37%) | 3 (10%) | 3 (10%) |  |

RN = Registered Nurse

LPN = Licensed Practical Nurse

MA = Medical Assistant

PSA = Patient Services Associate

**Table F.2.** Comparative analysis of provider survey characteristics between intervention site and control sites before and after implementation

|  | **Intervention Site** | | **Control Sites** | |  |  |
| --- | --- | --- | --- | --- | --- | --- |
|  | | | **Pre** | **Post** | **Pre** | **Post** |
| Provider survey characteristics | | | | | |  |
| Total, n | | 11 | 5 | 10 | 16 |  |
| Physician, n (%) | | 11 (100%) | 5 (100%) | 8 (80%) | 16 (100%) |  |
| APP, n (%) | |  |  | 2 (20%) |  |  |
| Time in role | | | | | |  |
| 0-2 years, n (%) | | 1 (9%) |  |  |  |  |
| 3-5 years, n (%) | |  | 1 (20%) | 5 (30%) | 3 (31%) |  |
| 6-10 years, n (%) | | 3 (27%) | 1 (20%) |  | 3 (19%) |  |
| 11-20, n years (%) | |  |  | 3 (30%) | 2 (12%) |  |
| More than 20 years, n (%) | | 7 (64%) | 3 (60%) | 4 (40%) | 6 (38%) |  |
| Time at clinic | | | | | |  |
| 0-2 years, n (%) | | 2 (18%) |  |  | 1 (6%) |  |
| 3-5 years, n (%) | |  | 1 (20%) | 5 (50%) | 7 (44%) |  |
| 6-10 years, n (%) | | 2 (18%) | 2 (40%) | 2 (20%) | 4 (25%) |  |
| 11-20 years, n (%) | | 2 (18%) |  | 2 (20%) | 1 (6%) |  |
| More than 20 years, n (%) | | 5 (46%) | 2 (40%) | 1 (10%) | 3 (19%) |  |

APP = Advanced Practice Provider

**Table F.3.** Responses to the question: ^b^*“The following statements are about portal messaging and in-basket workload.”* Evaluation of workload factors related to portal messages, presented as means with standard deviations (SD), deltas, difference-in-differences (DID), and 95% confidence intervals (CI). All assessed on a 1-5 Likert Scale.

|  | **Intervention Site** | | | **Control Sites** | | |  |
| --- | --- | --- | --- | --- | --- | --- | --- |
|  | **Pre** | **Post** | **Delta** | **Pre** | **Post** | **Delta** | **DID** |
| **^b^Workload** | | | | | | | |
| There is high variability in how providers want types of portal messages handled | 2.1 (1.0) | 2.3 (0.8) | -0.2 (-1.2, 0.9) | 2.2 (1.2) | 2.1 (1.0) | 0.1 (-0.5, 0.7) | -0.3 (-1.5, 0.9) |
| Portal messages routed back to my box as an FYI (with no further action needed from me) is appropriate and expected | 3.7 (1.5) | 3.5 (1.4) | 0.2 (-1.0, 1.5) | 3.0 (1.4) | 2.8 (1.2) | 0.2 (-0.5, 0.9) | 0.0 (-1.4, 1.4) |
| When portal messages are routed to my pool’s box and another pool or person, it is less clear to me where the responsibility of the message lies | 3.5 (1.0) | 3.1 (1.6) | 0.4 (-0.6, 1.5) | 2.7 (1.2) | 2.2 (1.0) | 0.5 (-0.1, 1.1) | -0.1 (-1.3, 1.2) |
| Portal messages routed back to a provider or another pool as an FYI (with no further action needed from them) is appropriate and expected | 2.8 (1.4) | 2.1 (1.1) | 0.7 (-0.3, 1.7) | 2.4 (1.2) | 2.1 (0.9) | 0.3 (-0.3, 0.9) | 0.4 (-0.8, 1.6) |
| At my clinic’s current level of staffing, I feel I have the bandwidth and time to address portal messages completely and to the top of my license | 2.7 (1.2) | 3.6 (1.5) | -0.9 (-2.2, 0.4) | 3.5 (1.4) | 3.0 (1.3) | 0.5 (-0.3, 1.2) | -1.4 (-2.9, 0.1) |
| When the clinic is short-staffed, I do not have time to complete the chart review or pre-work that I would like to before routing messages | 2.5 (1.0) | 3.2 (1.2) | -0.8 (-1.8, 0.2) | 2.4 (1.1) | 2.1 (0.9) | 0.2 (-0.3, 0.8) | -1.0 (-2.1, 0.1) |
| I can answer all portal messages routed to my team’s in-box during a normal 40-hour work week (if a 1.0 FTE) | 2.6 (0.9) | 3.1 (1.6) | -0.5 (-1.7, 0.7) | 3.4 (1.4) | 2.8 (1.2) | 0.6 (-0.1, 1.3) | -1.1 (-2.5, 0.3) |

**Table F.4.** Response to the questions: ^a^*“The following statements are about portal messaging and in-basket workload. Please indicate the frequency in which you typically encounter these situations.”* ^b^“*I route messages to both a provider and pool for the following reasons*.” Frequency and dual routing practices in portal messaging, presented as means with standard deviations (SD), deltas, difference-in-differences (DID), and 95% confidence intervals (CI). Responses ranked from most to least common.

|  | **Intervention Site** | | | **Control Sites** | | | |  |
| --- | --- | --- | --- | --- | --- | --- | --- | --- |
|  | **Pre** | **Post** | **Delta** | **Pre** | **Post** | **Delta** | | **DID** |
| **^a^Frequency** | | | | | | | | |
| I route portal message encounters to multiple people at once, mean (sd) [1-5 Likert] | 4.2 (1.5) | 1.8 (1.6) | 2.3 (0.7, 4.0) | 3.6 (1.6) | 3.0 (1.7) | 0.6 (-0.3, 1.4) | 1.8 (-0.1, 3.6 | |
| When portal messages seem urgent, I will route it to multiple providers and/or pools to ensure it is handled quickly | 3.4 (1.9) | 2.0 (1.5) | 1.4 (-0.6, 3.3) | 4.5 (1.7) | 4.0 (2.1) | 0.4 (-0.6, 1.4) | | 0.9 (-1.2, 3.1) |
| When there are too many portal messages in the in-basket competing with other work demands, I route them onwards to be addressed | 4.9 (2.0) | 5.2 (1.6) | -0.3 (-2.1, 1.6) | 4.0 (1.9) | 5.1 (1.7) | -1.1 (-2.0, -0.1) | | 0.8 (-1.3, 2.9) |
| Portal messaging interrupts my in-office clinic duties and patient care | 4.4 (2.0) | 3.3 (2.6) | 1.1 (-0.9, 3.1) | 2.7 (1.9) | 3.5 (1.8) | -0.8 (-1.9, 0.3) | | 1.9 (-0.4, 4.1) |
| **^b^Dual routing** | | | | | | | | |
| Urgent Message | 1.5 (0.8) | 1.0 (0.0) | 0.5 (-0.2, 1.2) | 1.6 (0.8) | 1.4 (0.7) | 0.2 (-0.2, 0.6) | | 0.3 (-0.5, 1.1) |
| Unsure who can handle the message the best | 2.1 (0.6) | 2.2 (0.5) | -0.1 (-0.8, 0.5) | 2.3 (0.8) | 2.4 (0.6) | -0.1 (-0.5, 0.3) | | -0.1 (-0.8, 0.7) |
| Provider should be included on most messages to ensure there nothing else to add | 2.4 (0.9) | 2.8 (0.5) | -0.4 (-1.1, 0.4) | 2.1 (0.7) | 2.2 (0.8) | -0.1 (-0.6, 0.3) | | -0.2 (-1.1, 0.6) |

**Table F.5.** Response to the questions: “*What types of things limit your ability to respond to portal messages within 48 hours?” ^b^“How do you prioritize your in-basket work? Rank from highest priority to lowest priority.”* Factors impacting message routing and inbox prioritization practices, presented as means with standard deviations (SD), deltas, difference-in-differences (DID), and 95% confidence intervals (CI). Responses ranked from most to least frequent.

|  | **Intervention Site** | | **Control Sites** | | | | |  |
| --- | --- | --- | --- | --- | --- | --- | --- | --- |
|  | **Pre** | **Post** | **Delta** | **Pre** | **Post** | | **Delta** | **DID** |
| **^a^Limit turnaround** | | | | | | | | |
| In-clinic patient care duties | 1.4 (0.7) | 2.0 (1.3) | -0.6 (-2.6, 1.4) | 2.9 (2.4) | 3.0 (2.1) | | -0.0 (-1.1, 1.0) | -0.5 (-2.8, 1.8) |
| Not enough information in initially routed message | 3.7 (1.7) | 3.4 (1.8) | 0.3 (-1.2, 1.8) | 3.3 (1.4) | 3.0 (1.6) | | 0.3 (-0.5, 1.1) | 0.0 (-1.7, 1.7) |
| Too many message in one day | 3.7 (1.5) | 3.5 (1.4) | 0.2 (-1.0, 1.5) | 3.0 (1.4) | 2.8 (1.2) | 0.2 (-0.5, 0.9) | | 0.0 (-1.4, 1.4) |
| Messages are not routed to me same day | 4.2 (1.5) | 4.6 (2.3) | -0.4 (-2.2, 1.4) | 3.7 (1.8) | 3.9 (2.0) | | -0.2 (-1.2, 0.8) | -0.2 (-2.3, 1.9) |
| Clerical work like faxes and forms | 4.7 (1.7) | 3.9 (2.0) | 0.8 (-0.9, 2.5) | 4.5 (1.9) | 5.0 (1.6) | | -0.5 (-1.4, 0.4) | 1.3 (-0.6, 3.3) |
| Issue is not appropriate for the portal | 6.1 (1.1) | 5.8 (0.9) | 0.4 (-1.3, 2.0) | 4.9 (1.7) | 4.6 (2.1) | | 0.3 (-0.6, 1.3) | 0.0 (-1.9, 1.9) |
| Unsure how to accomplish the task in the EHR (Technical knowledge gap) | 6.7 (1.5) | 7.4 (0.5) | -0.7 (-1.7, 0.3) | 7.2 (0.7) | 6.9 (1.2) | | 0.4 (-0.2, 0.9) | -1.1 (-2.2, 0.0) |
| Ambiguous routing | 6.9 (1.8) | 6.1 (2.5) | 0.8 (-1.1, 2.6) | 6.5 (1.7) | 6.6 (1.9) | | -0.1 (-1.1, 0.9) | 0.9 (-1.2, 2.9) |
| **^b^Prioritize inbox** |  |  |  |  |  | |  |  |
| Call Encounters | 1.5 (0.7) | 1.6 (0.9) | -0.2 (-0.9, 0.6) | 1.6 (0.9) | 1.6 (0.7) | | 0.1 (-0.3, 0.5) | -0.3 (-1.1, 0.6) |
| Medication Refills | 4.6 (1.6) | 3.2 (1.7) | 1.4 (-0.0, 2.8) | 3.3 (1.5) | 3.3 (1.5) | | -0.0 (-0.8, 0.8) | 1.4 (-0.2, 3.0) |
| Portal Messages | 2.3 (1.2) | 2.8 (1.4) | -0.5 (-1.5, 0.5) | 2.5 (1.0) | 2.3 (0.9) | | 0.2 (-0.4, 0.7) | -0.6 (-1.7, 0.5) |
| Result Messages from Providers | 3.6 (1.4) | 4.0 (1.2) | -0.4 (-1.7, 1.0) | 3.8 (1.6) | 3.9 (1.5) | | -0.0 (-0.8, 0.7) | -0.3 (-1.9, 1.2) |
| Faxes/Forms | 4.3 (1.2) | 5.2 (0.7) | -1.0 (-2.1, 0.2) | 4.8 (1.4) | 4.8 (1.2) | | -0.0 (-0.7, 0.6) | -1.0 (-2.3, 0.4) |
| Message Non-Encounters | 4.7 (1.2) | 4.1 (1.9) | 0.6 (-0.4, 1.6) | 5.0 (0.9) | 5.1 (1.0) | | -0.2 (-0.7, 0.4) | 0.8 (-0.4, 1.9) |

**Table F.6.** Response to the questions: ^a^“*The following questions touch on work-life balance.”* “*What factors about portal messages contribute to your stress and burnout?”* Factors impacting message routing and inbox prioritization practices, presented as means with standard deviations (SD), deltas, difference-in-differences (DID), and 95% confidence intervals (CI). Responses ranked from highest to lowest contributing factors.

|  | **Intervention Site** | | | **Control Sites** | | |  |
| --- | --- | --- | --- | --- | --- | --- | --- |
|  | **Pre** | **Post** | **Delta** | **Pre** | **Post** | **Delta** | **DID** |
| **^a^Burnout** | | | | | | | |
| Clerical burden contributes to my feelings of burnout or stress | 2.4 (0.9) | 2.4 (1.6) | -0.0 (-1.1, 1.1) | 2.0 (1.1) | 2.4 (1.2) | -0.4 (-1.0, 0.2) | 0.4 (-0.9, 1.6) |
| Portal messages contribute to my feelings of burnout or stress | 2.1 (1.0) | 2.6 (1.5) | -0.5 (-1.5, 0.4) | 2.1 (0.9) | 2.0 (0.9) | 0.1 (-0.5, 0.6) | -0.6 (-1.7, 0.5) |
| Current staffing shortages contribute to my feelings of burnout or stress | 1.7 (0.8) | 1.4 (0.7) | 0.4 (-0.6, 1.3) | 1.6 (1.2) | 1.6 (1.1) | 0.0 (-0.5, 0.6) | 0.3 (-0.8, 1.5) |
| **^b^Rank stress** | | | | | | | |
| Patient expectations in speed of response | 2.8 (2.1) | 2.9 (1.4) | -0.1 (-1.6, 1.4) | 2.3 (1.6) | 2.1 (1.4) | 0.2 (-0.7, 1.0) | -0.2 (-2.0, 1.5) |
| Patient expectations for what can be managed on the portal | 3.8 (2.0) | 2.6 (1.6) | 1.2 (-0.4, 2.8) | 2.7 (1.5) | 2.8 (1.8) | -0.2 (-1.1, 0.7) | 1.3 (-0.5, 3.2) |
| Confusion generated by multiple encounters/messages for the same patient issue | 2.7 (1.6) | 4.2 (2.3) | -1.6 (-3.3, 0.2) | 4.4 (1.9) | 3.9 (1.6) | 0.5 (-0.5, 1.4) | -2.0 (-4.0, -0.1) |
| Variability in provider expectations about portal message handling | 4.8 (1.9) | 5.5 (1.4) | -0.7 (-2.2, 0.8) | 4.8 (1.6) | 5.3 (1.5) | -0.5 (-1.3, 0.3) | -0.2 (-1.9, 1.5) |
| Provider communication responses for portal handling | 5.4 (1.4) | 5.1 (1.9) | 0.3 (-0.9, 1.5) | 5.4 (1.2) | 5.2 (1.2) | 0.2 (-0.5, 0.9) | 0.1 (-1.3, 1.5) |
| Delays in providers addressing portal messages once routed to them | 5.5 (1.6) | 5.4 (2.8) | 0.1 (-1.9, 2.1) | 4.5 (1.8) | 4.6 (2.2) | -0.0 (-1.1, 1.1) | 0.2 (-2.1, 2.4) |
| Lack of training or protocols to guide me on how to manage certain messages expected of me | 6.7 (2.1) | 6.4 (2.3) | 0.3 (-1.4, 2.0) | 7.5 (1.4) | 7.0 (1.9) | 0.5 (-0.4, 1.5) | -0.2 (-2.1, 1.8) |
| Balancing addressing portal messages while engaged in in-clinic duties | 4.3 (2.8) | 3.9 (2.5) | 0.4 (-2.0, 2.9) | 4.4 (2.6) | 5.1 (2.5) | -0.7 (-2.0, 0.7) | 1.1 (-1.7, 3.9) |

**Table F.7.** Responses to the question: *“The following statements are about portal messaging and in-basket workload.”* Evaluation of workload factors related to portal messages, presented as means with standard deviations (SD), deltas, difference-in-differences (DID), and 95% confidence intervals (CI). All assessed on a 1-7 Likert Scale.

|  | **Intervention Site** | |  | **Control Sites** | |  |  |
| --- | --- | --- | --- | --- | --- | --- | --- |
|  | **Pre** | **Post** | **Delta** | **Pre** | **Post** | **Delta** | **DID** |
| **Agreement** |  |  |  |  |  |  |  |
| Use of portal messages with patients has a positive impact on patient care | 2.0 (0.7) | 1.9 (0.7) | 0.1 (-1.1, 1.3) | 2.8 (1.4) | 2.3 (0.8) | 0.4 (-0.4, 1.3) | -0.4 (-1.8, 1.1) |
| Use of the portal and portal messages has a positive impact on patient satisfaction | 1.4 (0.5) | 1.9 (0.7) | -0.5 (-1.3, 0.3) | 2.1 (0.8) | 2.1 (0.7) | -0.0 (-0.6, 0.6) | -0.5 (-1.5, 0.5) |
| The expectations of all clinical team member roles in responding to portal messages is clear | 4.0 (1.7) | 4.6 (1.2) | -0.6 (-2.6, 1.4) | 4.4 (1.5) | 4.6 (1.6) | -0.1 (-1.5, 1.2) | -0.5 (-2.9, 2.0) |
| I can answer all of my portal messages during my standard working hours (e.g., 40 hours per 1.0 FTE) | 4.5 (1.7) | 4.9 (1.5) | -0.4 (-1.9, 1.2) | 5.4 (1.3) | 5.9 (0.3) | -0.5 (-1.6, 0.6) | 0.1 (-1.8, 2.1) |
| The majority of portal messages I am routed require physician input another staff member cannot address | 3.3 (0.6) | 4.5 (1.1) | -1.2 (-2.7, 0.4) | 4.0 (1.4) | 4.4 (1.1) | -0.4 (-1.4, 0.6) | -0.8 (-2.6, 1.1) |
| The majority of portal messages I am routed contain enough info that I can successfully manage them without much back and forth with patients | 3.2 (1.3) | 4.0 (1.2) | -0.8 (-2.3, 0.8) | 4.1 (1.5) | 4.2 (1.0) | -0.1 (-1.1, 1.0) | -0.7 (-2.6, 1.2) |

**Table F.8**. Responses to the question: ^a^*“The following statements are about portal messaging and in-basket workload.”* *^b^”The following statements are about portal message workflo*w.” Evaluation of workload and workflow factors related to portal messages, presented as means with standard deviations (SD), deltas, difference-in-differences (DID), and 95% confidence intervals (CI). All assessed on a 1-7 Likert Scale.

|  |  | **Intervention Site** | | |  | **Control Sites** | | | |  |  |  |
| --- | --- | --- | --- | --- | --- | --- | --- | --- | --- | --- | --- | --- |
|  | **Pre** | | **Post** | **Delta** | | | **Pre** | **Post** | **Delta** | | | **DID** |
| ^a^**Workload** |  | |  |  | | |  |  |  | | |  |
| How much time outside of your standard working hours do you spend addressing portal messages per week? | 3.6  (1.4) | | 2.6  (1.5) | 1.0  (-0.5, 2.5) | | | 3.2  (1.3) | 3.7  (1.4) | -0.5  (-1.6, 0.6) | | | 1.5  (-1.1, 4.1) |
| The following questions touch on work-life balance - Clerical burden contributes to my feelings of burnout and stress | 6.4  (0.7) | | 5.6  (2.2) | 0.7  (-0.3, 1.8) | | | 6.8  (0.7) | 6.4  (0.4) | 0.4  (-0.3, 1.9) | | | 0.3  (-1.4, 2.1) |
| The following questions touch on work-life balance - Portal messages contribute to my feelings of burnout and stress | 6.0  (1.0) | | 5.6  (2.2) | 0.4  (-1.2, 2.0) | | | 5.7  (0.8) | 6.3  (2.1) | 0.5  (-0.6, 1.7) | | | -0.1  (-2.6, 2.9) |
| ^b^**Workflow** |  | |  |  | | |  |  |  | | |  |
| Thinking of the last month, how often are the following pended appropriately with the necessary information before they are routed to you? – Orders | 2.5  (0.8) | | 2.2  (1.1) | 0.3  (-0.7, 1.4) | | | 2.4  (1.0) | 1.8  (0.8) | 0.6  (-1.4, 0.1) | | | 1.0  (-2.8, 1.0) |
| Thinking of the last month, how often are the following pended appropriately with the necessary information before they are routed to you? - Prescription Refills | 2.9  (0.7) | | 2.8  (1.3) | 0.1  (-0.9, 1.1) | | | 3.5  (0.8) | 3.2  (0.9) | -0.3  (-1.0, 0.4) | | | 0.4  (-1.3, 2.1) |
| Thinking of the last month, how often are the following pended appropriately with the necessary information before they are routed to you? – Patient Portal Messages | 2.1  (0.8) | | 1.8  (0.9) | 0.3  (-0.7, 1.2) | | | 2.3  (0.9) | 2.2  (0.6) | -0.1  (-0.8, 0.7) | | | 0.3  (-1.3, 2.0) |
| Thinking of the last month, how often are the following pended appropriately with the necessary information before they are routed to you? - Telephone Encounters | 2.6  (0.8) | | 2.0  (1.2) | 0.6  (-0.3, 1.6) | | | 2.4  (1.0) | 2.3  (0.7) | -0.1  (-0.8, 0.7) | | | 0.7  (-1.0, 2.4) |
| When a portal message is sent following up on a question about a visit or diagnostics done by a non-PCP physician or APP of the practice it should be routed to? | 2.9  (0.5) | | 2.0  (0.7) | 0.9  (0.4, 1.4) | | | 2.2  (0.3) | 2.1  (0.4) | -0.1  (-0.5, 0.3) | | | -1.0  (0.1 1.9) |

**Table F.9.** Responses to the question: *“How quickly should the following types of patient portal messages be answered (within business days and hours : 7am - 5pm , Monday - Friday ) ?* Timeliness of Response to Patient Requests - Percentage Distribution by Response Time and Request Type.

|  | **Intervention Site** | | | **Control Site** | |  | **Intervention Site** | | | **Control Site** | |
| --- | --- | --- | --- | --- | --- | --- | --- | --- | --- | --- | --- |
|  | | **Pre** | **Post** | **Pre** | **Post** |  | | **Pre** | **Post** | **Pre** | **Post** |
| **Medication Refill Requests** | | | | | | **Personal Updates** | | | | |  |
| Before the end of the business day | | 27% | 0% | 0% | 0% | Before the end of the business day | | 37% | 0% | 0% | 0% |
| Within 24 hours | | 36% | 20% | 31% | 30% | Within 24 hours | | 9% | 0% | 10% | 12% |
| 24-48 hours | | 37% | 60% | 57% | 50% | 24-48 hours | | 27% | 60% | 30% | 25% |
| 49-72 hours | | 0% | 20% | 6% | 20% | 49-72 hours | | 18% | 20% | 50% | 63% |
| Within 1 week | | 0% | 0% | 6% | 0% | Within 1 week | | 9% | 20% | 10% | 0% |
| **Medication Questions** | | | | | | **Mental Health Concerns** | |  |  |  |  |
| Before the end of the business day | | 27% | 0% | 0% | 0% | Before the end of the business day | | 64% | 20% | 20% | 19% |
| Within 24 hours | | 18% | 40% | 10% | 31% | Within 24 hours | | 27% | 20% | 0% | 19% |
| 24-48 hours | | 46% | 60% | 50% | 37% | 24-48 hours | | 9% | 60% | 60% | 44% |
| 49-72 hours | | 9% | 0% | 30% | 19% | 49-72 hours | | 0% | 0% | 10% | 12% |
| Within 1 week | | 0% | 0% | 30% | 13% | Within 1 week | | 0% | 0% | 10% | 6% |
| **Post visit follow up** | | | | | | **Schedule Requests** | |  |  |  |  |
| Before the end of the business day | | 9% | 0% | 0% | 0% | Before the end of the business day | | 9% | 0% | 0% | 0% |
| Within 24 hours | | 46% | 20% | 10% | 0% | Within 24 hours | | 37% | 0% | 10% | 6% |
| 24-48 hours | | 36% | 80% | 20% | 44% | 24-48 hours | | 9% | 60% | 30% | 25% |
| 49-72 hours | | 9% | 0% | 50% | 37% | 49-72 hours | | 36% | 40% | 50% | 25% |
| Within 1 week | | 0% | 0% | 20% | 19% | Within 1 week | | 9% | 0% | 10% | 44% |
| **Question about new symptoms** | |  |  |  |  | **Administrative Issues and Forms** | |  |  |  |  |
| Before the end of the business day | | 46% | 0% | 0% | 13% | Before the end of the business day | | 0% | 0% | 0% | 0% |
| Within 24 hours | | 36% | 20% | 10% | 37% | Within 24 hours | | 0% | 0% | 0% | 0% |
| 24-48 hours | | 0% | 80% | 50% | 31% | 24-48 hours | | 36% | 20% | 0% | 12% |
| 49-72 hours | | 9% | 0% | 30% | 19% | 49-72 hours | | 18% | 40% | 10% | 13% |
| Within 1 week | | 9% | 0% | 10% | 0% | Within 1 week | | 46% | 40% | 90% | 75% |
| **Referral Requests** | |  |  |  |  |  | |  |  |  |  |
| Before the end of the business day | | 37% | 0% | 0% | 0% |  | |  |  |  |  |
| Within 24 hours | | 27% | 60% | 0% | 0% |  | |  |  |  |  |
| 24-48 hours | | 18% | 40% | 10% | 12% |  | |  |  |  |  |
| 49-72 hours | | 18% | 0% | 20% | 25% |  | |  |  |  |  |
| Within 1 week | | 0% | 0% | 70% | 63% |  | |  |  |  |  |

**Table F.10.** Responses to the question: *“What types of things limit your ability to respond to portal messages within 48 hours?”* Perceived barriers to timely Handling of in-basket messages, presented as means with standard deviations (SD), deltas, difference-in-differences (DID), and 95% confidence intervals (CI). Responses ranked from most to least important.

| **Intervention Site** | | | | **Control Sites** | | | |
| --- | --- | --- | --- | --- | --- | --- | --- |
|  | **Pre** | **Post** | **Delta** | **Pre** | **Post** | **Delta** | **DID** |
| **Limit 48 Hours** |  |  |  |  |  |  |  |
| Seeing patients that day | 4.0 (1.4) | 2.8 (2.2) | 1.2 (-0.9, 3.3) | 2.2 (1.6) | 2.3 (1.7) | -0.1 (-1.6, 1.4) | 1.3 (-1.3, 3.9) |
| Not enough information in initially routed message | 3.2 (1.7) | 3.4 (1.2) | -0.1 (-1.5, 1.3) | 3.2 (1.0) | 2.7 (1.3) | 0.5 (-0.5, 1.5) | -0.6 (-2.3, 1.1 |
| Too many messages in one day | 1.8 (1.0) | 2.8 (1.3) | -1.1 (-2.8, 0.7) | 3.0 (1.8) | 2.6 (1.2) | 0.4 (-0.8, 1.6) | -1.5 (-3.6, 0.6) |
| Messages are not routed to me same day | 2.2 (1.0) | 2.5 (1.6) | -0.2 (-1.9, 1.5) | 2.9 (1.1) | 3.2 (1.7) | -0.3 (-1.5, 0.8) | 0.1 (-1.9, 2.2) |
| Issue is not appropriate for the portal | 3.8 (1.3) | 4.9 (1.0) | -1.2 (-2.6, 0.3) | 4.1 (1.5) | 4.5 (0.8) | -0.4 (-1.5, 0.6) | -0.7 (-2.5, 1.1) |
| Unsure how to accomplish the task in EHR (technical knowledge gap) | 6.2 (0.5) | 5.7 (1.8) | 0.5 (-0.7, 1.7) | 6.8 (0.4) | 6.8 (0.4) | -0.0 (-0.8, 0.8) | 0.5 (-0.9, 2.0) |
| Ambiguous routing (multiple providers/pools included, unclear if it is my responsibility to manage) | 6.8 (0.5) | 5.9 (1.3) | 0.8 (-0.3, 2.0) | 5.9 (0.9) | 5.9 (0.7) | -0.0 (-0.8, 0.8) | 0.9 (-0.5, 2.3) |

**Table F.11.** Responses to the question: *“What types of patient portal messages take the most time (as currently handled)?* Time consumption ratings for various In-basket message categories, presented as means with standard deviations (SD), deltas, difference-in-differences (DID), and 95% confidence intervals (CI). Responses ranked from most to least time consuming.

| **Intervention Site** | | | | **Control Sites** | | | |
| --- | --- | --- | --- | --- | --- | --- | --- |
|  | **Pre** | **Post** | **Delta** | **Pre** | **Post** | **Delta** | **DID** |
| **Time Consuming** |  |  |  |  |  |  |  |
| Medication Refill Requests | 6.6 (3.4) | 6.6 (2.3) | -0.0 (-2.4, 2.3) | 6.9 (1.7) | 6.9 (1.8) | 0.0 (-1.7, 1.8) | -0.1 (-3.0, 2.8) |
| Medication Questions | 3.8 (1.5) | 5.1 (1.3) | -1.3 (-2.7, 0.1) | 3.8 (1.2) | 4.8 (1.3) | -1.0 (-2.1, 0.0) | -0.2 (-2.0, 1.5) |
| Post-Visit Follow Up | 4.6 (1.8) | 4.9 (2.1) | -0.3 (-2.1, 1.5) | 5.2 (1.2) | 3.8 (1.5) | 1.4 (0.1, 2.8) | -1.8 (-4.0, 0.4) |
| Questions About New Symptoms | 2.4 (1.1) | 1.5 (0.7) | 0.9 (-0.5, 2.4) | 1.8 (1.8) | 1.4 (0.7) | 0.4 (-0.7, 1.5) | 0.5 (-1.2, 2.3) |
| Referral Requests | 5.8 (0.8) | 5.7 (1.5) | 0.1 (-1.5, 1.6) | 5.8 (1.2) | 5.1 (1.7) | 0.6 (-0.5, 1.8) | -0.6 (-2.5, 1.3) |
| Personal Updates | 7.8 (1.1) | 7.1 (1.6) | 0.7 (-1.4, 2.8) | 6.6 (2.3) | 7.1 (1.9) | -0.5 (-2.1, 1.0) | 1.2 (-1.4, 3.9) |
| Mental Health Concerns | 3.6 (2.4) | 4.3 (2.1) | -0.7 (-3.0, 1.6) | 3.4 (1.8) | 3.6 (2.4) | -0.2 (-1.9, 1.6) | -0.5 (-3.4, 2.4) |

**Table F.12.** Responses to the question: *“What types of patient portal messages cause the most stress/frustration?”* Stress ratings for various in-basket message categories, presented as means with standard deviations (SD), deltas, difference-in-differences (DID), and 95% confidence intervals (CI). Responses ranked from most to least stressful.

| **Intervention Site** | | | | | **Control Sites** | | | |
| --- | --- | --- | --- | --- | --- | --- | --- | --- |
|  | **Pre** | **Post** | | **Delta** | **Pre** | **Post** | **Delta** | **DID** |
| **Stress** |  |  | |  |  |  |  |  |
| Medication Refill Requests | 7.8 (1.9) | 5.8 (2.8) | 1.9 (-1.1, 4.9) | | 6.1 (2.7) | 6.9 (2.0) | -0.8 (-2.9, 1.4) | 2.7 (-1.0, 6.4) |
| Medication Questions | 5.2 (1.0) | 5.5 (1.4) | -0.3 (-1.8, 1.2) | | 4.6 (1.2) | 5.2 (1.4) | -0.7 (-1.7, 0.4) | 0.4 (-1.5, 2.2) |
| Post-Visit Follow Up | 4.2 (2.1) | 5.4 (1.7) | -1.1 (-3.4, 1.2) | | 5.4 (1.9) | 4.1 (2.1) | 1.3 (-0.4, 2.9) | -2.4 (-5.2, 0.4) |
| Questions About New Symptoms | 3.5 (1.7) | 2.8 (2.0) | 0.7 (-1.7, 3.1) | | 3.0 (2.3) | 2.4 (1.7) | 0.6 (-1.2, 2.3) | 0.1 (-2.8, 3.1) |
| Referral Requests | 7.0 (0.8) | 5.1 (2.5) | 1.9 (-0.3, 4.1) | | 5.5 (1.4) | 4.9 (1.9) | 0.6 (-0.9, 2.2) | 1.3 (-1.4, 4.0) |
| Personal Updates | 8.5 (0.6) | 7.7 (1.3) | 0.8 (-1.4, 2.9) | | 7.1 (2.6) | 7.7 (0.7) | -0.5 (-2.1, 1.0) | 1.3 (-1.4, 4.0) |
| Mental Health Concerns | 3.0 (2.3) | 4.6 (2.1) | -1.6 (-4.4, 1.1) | | 4.3 (2.4) | 4.2 (2.4) | 0.1 (-1.9, 2.1) | -1.7 (-5.1, 1.7) |

**Table G.1.** *Absolute pre-post differences for intervention and control sites and difference in differences.* *We report relative differences for these same measures in Table 4.*

|  | Intervention Site | | | Control Sites | | |  |
| --- | --- | --- | --- | --- | --- | --- | --- |
| Measure (per month) | Pre, mean (95% CI) | Post, mean (95% CI) | Difference  (95% CI) | Pre, mean (95% CI) | Post, mean (95% CI) | Difference  (95% CI) | DID  (95% CI) |
| Messages received per cFTE^1^ per month | 1,899  (1,493-2,414) | 1,037  (816-1,319) | -861.4  (1,329 to -393.8) | 1,384  (1,088-1,759) | 948  (746-1,205) | -435.4  (-797,6 to -73.3) | -425.9  (-1,017.4-165.5) |
| Messages sent per cFTE^2^ per month | 1,342  (1,269-1,420) | 954  (902-1,010) | -388.1  (-471.6 to -304.6) | 1,103  (1,044-1,166) | 935  (885-988) | -168.5  (-240.0 to -96.9) | -219.6  (-329.6 to -109.7) |
| % “Carbon Copy” messages^3^ (with more than 1 recipient), % | 4.4  (4.1-4.6) | 1.5  (1.4-1.7) | -2.9  (-3.1 to -2.6) | 1.1  (1.0-1.1) | 1.0  (0.9-1.0) | -0.1  (-0.2 to -0.0) | -2.7  (-3.1 to -2.4) |
| Number of messages per encounter | 4.3  (4.3-4.4) | 3.4  (3.3-3.5) | -0.9  (-1.0 to -0.8) | 4.2  (4.1-4.2) | 3.6  (3.6-3.7) | -0.5  (-0.6 to -0.5) | -0.4  (-0.5 to -0.3) |
| Messages to physicians per cFTE per month | 428  (395-463) | 319  (294-345) | -109.3  (-147.3 to -71.2) | 224  (207-242) | 194  (180-209) | -29.9  (-50.2 to -9.5) | -79.4  (-122.6 to -36.2) |
| Messages sent by physicians per cFTE^2^ per month | 65  (56-75) | 64  (56-74) | -0.4  (-12.2-11.4) | 96  (84-110) | 81  (71-93) | -15.1  (-30.2-0.0) | 14.7  (-4.5 to 33.9) |
| New encounters per cFTE per month | 421  (395-449) | 298  (279-318) | -123.2  (-153.3 to -93.0) | 328  (308-348) | 254  (239-271) | -73.1  (-95.9 to -50.3) | -50.1  (-87.9 to -12.3) |
| % encounters with physician involvement, % | 60.3  (59.3-61.3) | 59.9  (58.9-61.0) | -0.3  (-1.8-1.2) | 46.9  (46.4-47.4) | 50.6  (50.1-51.1) | 3.7  (3.0-4.4) | -4.0  (-5.7 to -2.4) |
| % encounters with a message sent by a physician, % | 11.9  (11.2-12.5) | 16.5  (15.7-17.3) | 4.6  (3.6-5.7) | 23.0  (22.6-23.4) | 24.6  (24.2-25.1) | 1.6  (1.0-2.2) | 3.0  (1.8-4.3) |
| % encounters triaged by physicians, % | 2.7  (2.3-3.1) | 9.6  (8.8-10.4) | 6.9  (6.0-7.8) | 1.4  (1.3-1.6) | 1.3  (1.2-1.5) | -0.1  (-0.3 to 0.1) | 7.0  (6.1-7.9) |

Abbreviations: CI = Confidence Interval, cFTE = Clinical Full Time Equivalent, DID = Difference in Differences

^1^ Total number of messages received by all clinic providers and staff per physician cFTE per month.

^2^ Excludes messages sent to patients.

^3^ Excludes messages sent to 1 physician and 1 resident; ^4^ Excluding physician-triaged by encounters.

**Table G.2.** Relative change for exploratory portal messaging metrics post-intervention compared to pre-intervention.

|  | **Intervention Site** | | | **Control Sites** | | |  |
| --- | --- | --- | --- | --- | --- | --- | --- |
| Measure (per month) | Pre, mean  (95% CI) | Post, mean  (95% CI) | RR  (95% CI) | Pre, mean (95% CI) | Post, mean (95% CI) | RR  (95% CI) | RRR  (95% CI) |
| Messages Received per cFTE^1^ | 1,899  (1,493-2,414) | 1,037  (816-1,319) | 0.55  (0.39-0.77) | 1,384  (1,088-1,759) | 948  (746-1,205) | 0.69  (0.49-0.96) | 0.80  (0.49-1.29) |
| Number of messages per encounter | 4.3  (4.3-4.4) | 3.4  (3.3-3.5) | 0.78  (0.77-0.80) | 4.2  (4.1-4.2) | 3.6  (3.6-3.7) | 0.87  (0.86-0.88) | 0.90  (0.87-0.92) |
| Messages to Physicians per cFTE | 428  (395-463) | 319  (294-345) | 0.74  (0.67-0.83) | 224  (207-242) | 194  (180-209) | 0.87  (0.78-0.97) | 0.86  (0.74-1.00) |
| Messages sent by Physicians per cFTE^2^ | 65  (56-75) | 64  (56-74) | 0.99  (0.81-1.22) | 96  (84-110) | 81  (71-93) | 0.84  (0.70-1.02) | 1.18  (0.89-1.56) |
| New Encounters per cFTE | 421  (395-449) | 298  (279-318) | 0.71  (0.65-0.78) | 328  (308-348) | 254  (239-271) | 0.78  (0.71-0.85) | 0.91  (0.80-1.03) |
| % encounters with a message sent by a physician^3,4^, % | 11.9  (11.2-12.5) | 16.5  (15.7-17.3) | 1.39  (1.29-1.50) | 23.0  (22.6-23.4) | 24.6  (24.2-25.1) | 1.07  (1.04-1.10) | 1.30  (1.20-1.41) |
| % encounters triaged by physicians (first non-patient sender), % | 2.7  (2.3-3.1) | 9.6  (8.8-10.4) | 3.58  (3.00-4.27) | 1.4  (1.3-1.6) | 1.3  (1.2-1.5) | 0.93  (0.81-1.08) | 3.83  (3.06-4.81) |

Abbreviations: cFTE = Clinical Full Time Equivalent, RR = Risk Ratio, RRR = Relative Risk Ratio

^1^ Total number of messages received by all clinic providers and staff per physician cFTE per month.

^2^ Excludes messages sent to patients.

^3^ Excludes messages sent to 1 physician and 1 resident

^4^ Excluding physician-triaged encounters.

**Table G.3.** *Summary of denominators used for reported rates****.***

| **Denominator** | **Total** | **Intervention Site** | | **Control Sites** | |
| --- | --- | --- | --- | --- | --- |
|  |  | **Pre** | **Post** | **Pre** | **Post** |
| Months | 6 | 3 | 3 | 3 | 3 |
| Physician cFTE per month | 35.9 | 5.0 | 6.0 | 29.4 | 31.3 |
| Messages | 342,990 | 36,641 | 25,055 | 118,817 | 162,477 |
| Encounters | 87,504 | 8,438 | 7,350 | 39,054 | 32,662 |
| Encounters with first message from patient | 65,042 | 5,826 | 5,127 | 28,276 | 25,813 |

Abbreviations: cFTE = clinical Full Time Equivalent (100% = 40 hours per week, 32 in clinic and 8 administrative).
